# Autophagy inhibitor facilitates gefitinib sensitivity *in vitro* and *in vivo* by activating mitochondrial apoptosis in triple negative breast cancer

**DOI:** 10.1371/journal.pone.0177694

**Published:** 2017-05-22

**Authors:** Zhaoyun Liu, Kewen He, Qinghua Ma, Qian Yu, Chenyu Liu, Isabella Ndege, Xinzhao Wang, Zhiyong Yu

**Affiliations:** 1 School of Medicine and Life Sciences, University of Jinan-Shandong Academy of Medical Sciences, Jinan, Shandong, China; 2 Department of Oncology, Shandong Cancer Hospital affiliated to Shandong University, Shandong Academy of Medical Sciences, Jinan, Shandong, China; 3 University of Kentucky College of Medicine, Lexington, Kentucky, United States of America; 4 Department of Biology, Winship Cancer Institute, Emory University, Atlanta, Georgia, United States of America; University of South Alabama, UNITED STATES

## Abstract

Epidermal growth factor receptor (EGFR) is over-expressed in about 50% of Triple negative breast cancers (TNBCs), but EGFR inhibitors have not been effective in treating TNBC patients. Increasing evidence supports that autophagy was related to drug resistance at present. However, the role and the mechanism of autophagy to the treatment of TNBC remain unknown. In the current study, we investigated the effect of autophagy inhibitor to gefitinib (Ge) in TNBC cells *in vitro* and *in* nude mice *vivo*. Our study demonstrated that inhibition of autophagy by 3-Methyladenine or bafilomycin A1 improved Ge’s sensitivity to MDA-MB-231 and MDA-MB-468 cells, as evidence from stronger inhibition of cell vitality and colony formation, higher level of G0/G1 arrest and DNA damage, and these effects were verified *in* nude mice *vivo*. Our data showed that the mitochondrial-dependent apoptosis pathway was activated in favor of promoting apoptosis in the therapy of Ge combined autophagy inhibitor, as the elevation of BAX/Bcl-2, Cytochrome C, and CASP3. These results demonstrated that targeting autophagy should be considered as an effective therapeutic strategy to enhance the sensitivity of EGFR inhibitors on TNBC.

## Introduction

Triple negative breast cancer (TNBC), characterized as estrogen receptor negative, progesterone receptor negative, and human epidermal growth factor receptor 2 negative, accounts for roughly 15%-20% of all breast cancer patients [1, 2]. Women with TNBC have a peak risk of recurrence and mortality within 3 to 5 years from the time of diagnosed due to its aggressive nature, and a median survival after recurrence of ~9 months [3, 4]. Because TNBC lacks an approved targeted therapy, the only remaining systemic treatment is chemotherapy, which has been reported to respond poorly [5, 6]. Therefore, novel therapies are urgently required to improve its prognosis. Epidermal growth factor receptor (EGFR), which is over-expressed in about 50% of TNBCs, is related to boosting tumor growth and increasing metastasis rates, and representing poor clinical outcomes [7, 8]. EGFR inhibitors are considered as a hopeful strategy for cancer therapy in diverse EGFR^+^ cancers clinically: Gefitinib (Ge, small molecules EGFR tyrosine kinases inhibitor), which inhibits cancer growth mainly through targeting the adenosine triphosphate binding sites in the cytoplasmic domain of EGFR, is widely applied in non-small-cell lung cancer (NSCLC); Cetuximab (monoclonal antibody) was approved in colorectal cancer, etc. Unfortunately, their efficacy for breast cancer is limited due to drug resistance [9]. Several lines of evidence suggested that EGFR targeted therapy could induce cytoprotective autophagy that was related to both innate and acquired drug resistance in different tumor cell lines [10–12].

Autophagy, an evolutionarily conserved lysosomes degradation process, degrades the cytosolic contents into essential components for the recycling and the rebuilding of cellular macromolecules [13]. It has been well acknowledged that the abnormal autophagic process responsible for drug resistance could emerge in cells stressed by targeted drugs, so autophagy inhibitor appeared to be a therapeutic approach for sensitizing target therapy [14–16]. Evidences reported that blockage of autophagy increased the sensitivity of chemotherapy, radiotherapy, and EGFR target therapy in NSCLC cells [17–19]. Chen *et al* reported that inhibiting the cytoprotective autophagy induced by gemcitabine enhanced apoptosis in TNBC cells [20]. Cufi *et al* found that autophagy was involved in HER2-targeted therapy in breast cancer, and was associated with drug resistance [21]. However, we found few report about EGFR target therapy and autophagy in TNBC cells.

Mitochondria, playing a central role of ATP generation, is the key mediator involved in the process of apoptosis [22]. When caspase protease was activated, it would digest numerous proteins which can result in cell death, such as, cleaved CASP3, one of the final players in the apoptosis signaling pathway [23, 24]. Usually, caspase can be activated by the mitochondrial, which is the main process in the induction of apoptosis [25]. When mitochondrial were damaged, mitochondrial outer membrane protein (MOMP) would be triggered. After the activation of MOMP, mitochondrial intermembrane space proteins, notably cytochrome C, would be released. Cytochrome C, did not only plays an essential role in mitochondrial ATP generation but also was vital for caspase activation following its release from mitochondria [26].

In this study, we found that autophagy inhibitor such as 3-MA and Baf.A facilitated the efficiency of Ge as evidence from cell proliferation inhibition by activating mitochondrial apoptosis in TNBC cells.

## Materials and methods

### Pharmacological reagents

Gefitinib (Ge) was purchased from Tocris Bioscience Company (Bristol, UK). 3-Methyladenine (3-MA) and bafilomycin A1 (Baf.A) were purchased from Selleckchem (Houston, USA). Ge, 3-MA and Baf.A were dissolved in 100% dimethyl sulfoxide (DMSO; Fisher Scientific, Pittsburgh, PA, USA). In all cases of cell treatment, the final DMSO concentration never exceeded 0.2% in the culture medium. Stock solutions of all drugs were stored at −20°C.

### Cell culture and treatment

TNBC derived cells lines (MDA-MB-468 and MDA-MB-231) were purchased from the Cell Bank of Shanghai Institute of Cell Biology, Chinese Academy of Sciences, and were cultured in DMEM media (High glucose, HyClone Company, UT, United States) supplemented with 10% fetal bovine serum (Sijiqing Company, Hangzhou, China) and 100 units/ml antibiotics (penicillin/streptomycin, Gibco/Invitrogen) in a humidified atmosphere of 5% CO_2_ at 37°C. Cells were seeded in cell culture plates and allowed to adhere overnight, subsequently subjected to DMSO (0.2%), 3-MA (10 mM), Baf.A (1nM), Ge (5 μM), Ge (5 μM) +3-MA (10 mM) and Ge (5 μM) +Baf.A (1nM) treatment for 48 hours, respectively.

### Immunofluorescence (IF)

Cells were seeded on Glass Bottom Cell Culture Dishes (NEST, 801007) and then the cells were exposed to treatments as indicated above for 48 hours. Cells were seeded with 4% paraformaldehyde, incubated with 0.1% Triton X-100 for 30 min, and then incubated with anti-LC3 antibodies (1:200) (CST, 2775S) overnight at 4°C. Next, cells were incubated with Cy3-labeled Goat Anti-Rabbit IgG (H+L) (1:200) (Beyotime, A0516) for 1 hour, washed with PBS. Then 4', 6-diamidino-2-phenylindole (DAPI) (Biosharp, C1002) were used to stain nuclei. Microscopy was done on a confocal laser microscopy (OLYMPUS, BX53).

### CCK8 assay

The MDA-MB-468 and MDA-MB-231 cells were respectively plated in 96-well plates. After treatment with the indicated concentration (0, 1.25, 2.5, 5, 10 and 20 μM) of Ge in present of 3-MA/Baf.A or not for 48 hours, CCK8 was added to each well, followed by incubation at 37°C in 5% CO_2_ for 2 hours. Absorbance (A) was measured on a Bio-Rad 680 microplate reader (Bio-rad 680, Bio-Rad Laboratories, Hercules, USA) at 570 nm, and the results were reported relative to a reference wavelength of 630 nm. The cell viability rate was calculated according to the following: Cell viability rate = (A^drug-treated^/A^DMSO^) × 100%. The experiment was repeated three times.

### Colony formation assay

The cells were plated in 6-well plate and exposed to above drugs, and then incubated at 37°C for 14 days. Then the cells were fixed with 4% paraformaldehyde and stained with crystal violet. The number of colonies (>50 cells) was counted. The colony formation rate was calculated with the following formula: Survival Fraction = (Clones/Cell numbers) × 100%.

### *In vivo* studies

All animal studies were approved by the Committee on the Ethics of Animal Experiments of the Shandong Cancer Hospital (Permit Number: SDTHEC-201503041). Mice were housed according to the guidelines outlined with full respect to the EU Directive 2010/63/EU for animal experimentation. Forty healthy BALB/c female nude mice (4–6 weeks old) were purchased from the HFK Bioscience Company (Beijing, China). The mice were fed with water and food in a specific environment maintained at 23±1°C. Animals were under isoflurane inhalation anesthesia when they were injected every time to minimize suffering. Approximately 1×10^7^ MDA-MB-468 cells in 100 μl PBS were subcutaneously inoculated into the left flank of nude mice. Then we placed animals in their cages to recover and monitored them until they were awake. Animals were monitored every day including vitality, mental state, and skin color. When thirty mice’s tumor xenografts had grown to nearly 100 mm^3^ in size, the mice were randomized into six treatment groups with 5 mice every group as follows: vehicle, 3-MA, Baf.A, Ge, Ge+3-MA, and Ge+Baf.A. 3-MA and Baf.A were injected intratumorally, Ge was administered via oral gavage. The combined treatment was the same as the single agent treatment. DMSO was administered to the vehicle-treated group. There were 4 mice sacrificed after the oral gavage, and we thought it may be resulted from the operation of the oral gavage. All mice with or without tumors were sacrificed under isoflurane inhalation anesthesia 15 days later after the drug treatment, and the tumors were separated after completion of treatment. Size of local tumors were calculated by measuring length and width every two days using a caliper, and the tumor volume (TV) was calculated according to the formula: TV (mm^3^) = 1/2 × (length × square width).

### Analysis of the cell cycle distribution

The cells were respectively seeded in 6-well plates overnight. Then the cells were treated with the before mentioned treatment at indicated concentrations for 48 hours and then fixed in 70% of ethanol for 72 hours. After being washed twice with PBS, the cells were stained with propidium iodide (PI) for 30 min. Flow cytometric analysis was performed on the FACS Calibur (Becton Dickinson, USA). The data were analyzed using ModiFit software (Topsham, ME, USA).

### Western blot

The cells were plated and treated as described above for 48 hours, and then were lysed in cell lysis buffer (Beyotime, P0013, Beijing, China) supplemented with 0.5 mM phenylmethanesulfonyl fluoride (PMSF, Beyotime, ST506). The total cellular protein concentration was determined with a BCA Protein Assay Kit (Thermo Fisher Scientific Inc., 23227, Rockford, USA). The proteins were applied to sodium dodecyl polyacrylamide gel electrophoresis, and transferred onto a PVDF membrane (Millipore, Billerica, MA, USA). Then membranes were blocked with 5% evaporated skimmed milk for 1 hour and probed overnight at 4°C with the following primary antibodies: cleaved-CASP 3 (9664), Cytochrome C (4280), Phospho-Chk1 (2348), Phospho-Chk2 (2197), Phospho-ATM (5883), Phospho-Histone H2A.X (9718), BAX (2772), Bcl-2 (2870), (all 1:1000; Cell Signaling Technology, Danvers, MA, USA), antibody against ACTB (1:2000; Zsbio, sc-53142, Beijing, China), followed by incubation with horseradish peroxidase coupled secondary anti-mouse (Zsbio, ZB-2305) or anti-rabbit antibodies (Zsbio, ZB-2301) for 1 hour at room temperature. The protein bands were visualized using ECL blotting detection reagents (Beyotime, P0018), and developed and fixed onto x ray films. ACTB was served as a loading control.

### Ex vivo analysis of MDA-MB-468 xenografts tissue

The nude mice were euthanized before separation of tissue. Tumor tissues were fixed and prepared as 5 μm paraffin sections on microscope slides for hematoxylin-eosin staining and immunofluorescence, and they were dewaxed using routine techniques. The slides were incubated in antigen retrieval buffer and boiled for 10 min, then cooled to room temperature. After peroxidase blocking with 3% H_2_O_2_ for 15 min, specimens were blocked with goat serum (Solarbio, China) in phosphate-buffered saline (PBS) for 15 min. Then cleaved-CASP 3 (9664), (1:200; Cell Signaling Technology, Danvers, MA, USA) were carried out overnight at 4°C. After hematoxylin staining, dehydration, transparent, and sealing film, we observed the microscope slides at 400 × microscope.

### Statistical analysis

Data were analyzed using GraphPad Prism 6.02 (GraphPad Software, San Diego, CA, USA). Significant differences between two samples were conducted by t-test. All statistical significance was evaluated with data from at least three independent experiments. Data were presented as the mean ± SD. statistical tests employed at a significance level of 0.05 to determine whether a significant difference existed.

## Results

### Autophagy is activated by Ge, and inhibited by 3-MA or Baf.A in TNBC cells

**Fig 1:**
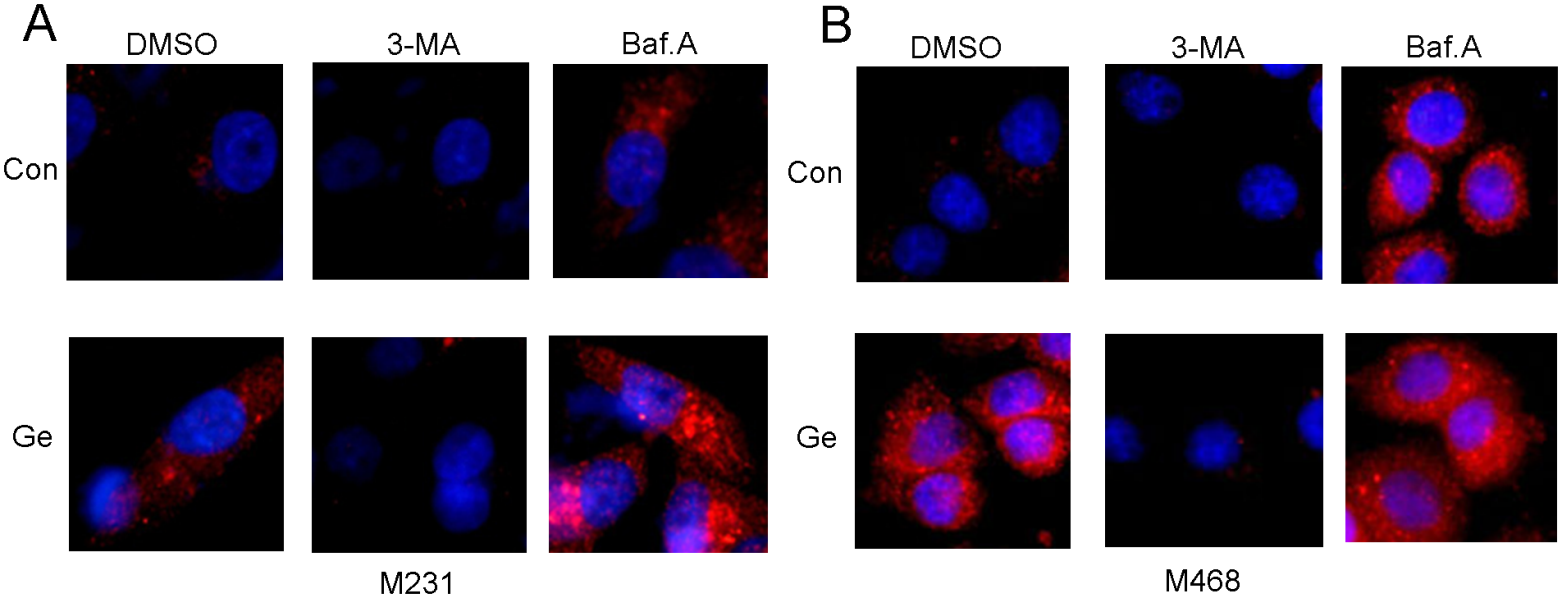
Autophagy is activated by Ge and inhibited by 3-MA or Baf.A in TNBC cells. Cells were exposed with 3-MA (10 mM) and Baf.A (1 nM) alone or with gefitinb (5 μM) for 48 hours, and the inhibitory were confirmed by immunofluorescence staining. LC3 puncta in the cells were detected by immunofluorescence under confocal laser microscopy in MDA-MB-231 (A) and MDA-MB-468 (B).

To evaluate Ge-induced autophagy in TNBC cells, we detected the LC3 expression with immunofluorescence staining in MDA-MB-468 and MDA-MB-231 cells after their treatment with Ge. As shown in Fig 1, we confirmed the inhibitory effect of 3-MA and Baf.A on Ge induced autophagy by monitoring the protein level of LC3 with or without presence of 3-MA or Baf.A. As expected, 3-MA (blocking the form of autophagosome) decreased the numerous of autophagosomes while Baf.A (blocking the fusion of lysosomes and autophagosome) lead to an accumulation of autophagosomes. Both 3-MA and Baf.A involved in the autophagy inhibited reaction. Taken together, our data indicated that autophagy was accompanied by Ge therapy, which might contribute to the resistance of Ge.

### Autophagy inhibitor facilitates cytotoxicity of Ge in TNBC cells *in vitro*

**Fig 2:**
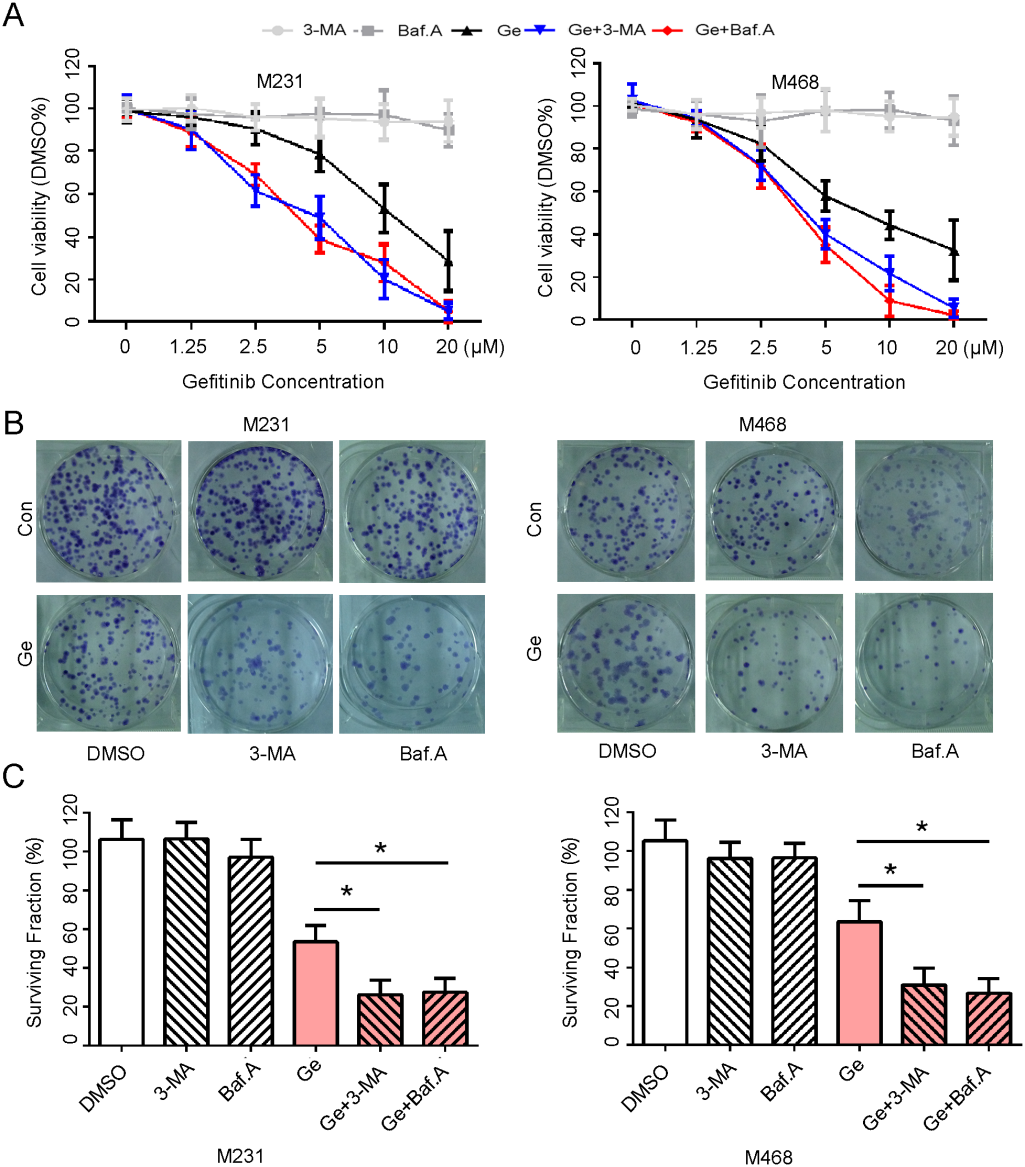
Autophagy inhibitor facilitates cytotoxicity of Ge in TNBC cells *in vitro*. **(A)** MDA-MB-231 and MDA-MB-468 cells were treated with 0, 1.25, 2.50, 5.00, 10.00, 20.00 μM Ge alone or combined with 3-MA (10 mM) or Baf.A (1 nM) respectively for 48 hours, DMSO acted as the control, and then subjected to CCK8 assay. Absorbance value was calculated and standardized to DMSO group. Three independent experiments were performed. **(B)** The above cells were treated with DMSO (0.2%), 3-MA (10 mM), Baf.A (1 nM), Ge (5 μM), Ge (5 μM) +3-MA (10 mM) and Ge (5 μM) +Baf.A (1 nM), DMSO acted as the control, and subjected to cell colony formation assay. **(C)** Cell surviving fraction were calculated and presented as mean ± SD; *p < 0.05. Three independent experiments were performed.

In order to detect the facilitation of autophagy inhibitor on Ge in breast cells, we employed and exposed MDA-MB-231 and MDA-MB-468 cell lines to treatment with control (Dimethyl Sulphoxide, DMSO), 3-MA, Baf.A, Ge, Ge+3-MA, Ge+Baf.A, respectively. As shown in Fig 2A, Ge inhibited the cell viability of MDA-MB-468 and MDA-MB-231 cells more potently when combined with 3-MA or Baf.A than Ge alone. In addition, a clonogenic assay was also performed and demonstrated that colony formation was suppressed significantly by combination of Ge and autophagy inhibitor, as evidenced by a lower clonogenic survival rate, whereas Ge alone slightly weaken colony formation in all detected cells (Fig 2B and 2C). These results supported that autophagy inhibitor combined with Ge enhanced the inhibition of cells in growth and colony formation.

### Autophagy inhibitor facilitates cytotoxicity of Ge in TNBC xenografts *in vivo*

**Fig 3:**
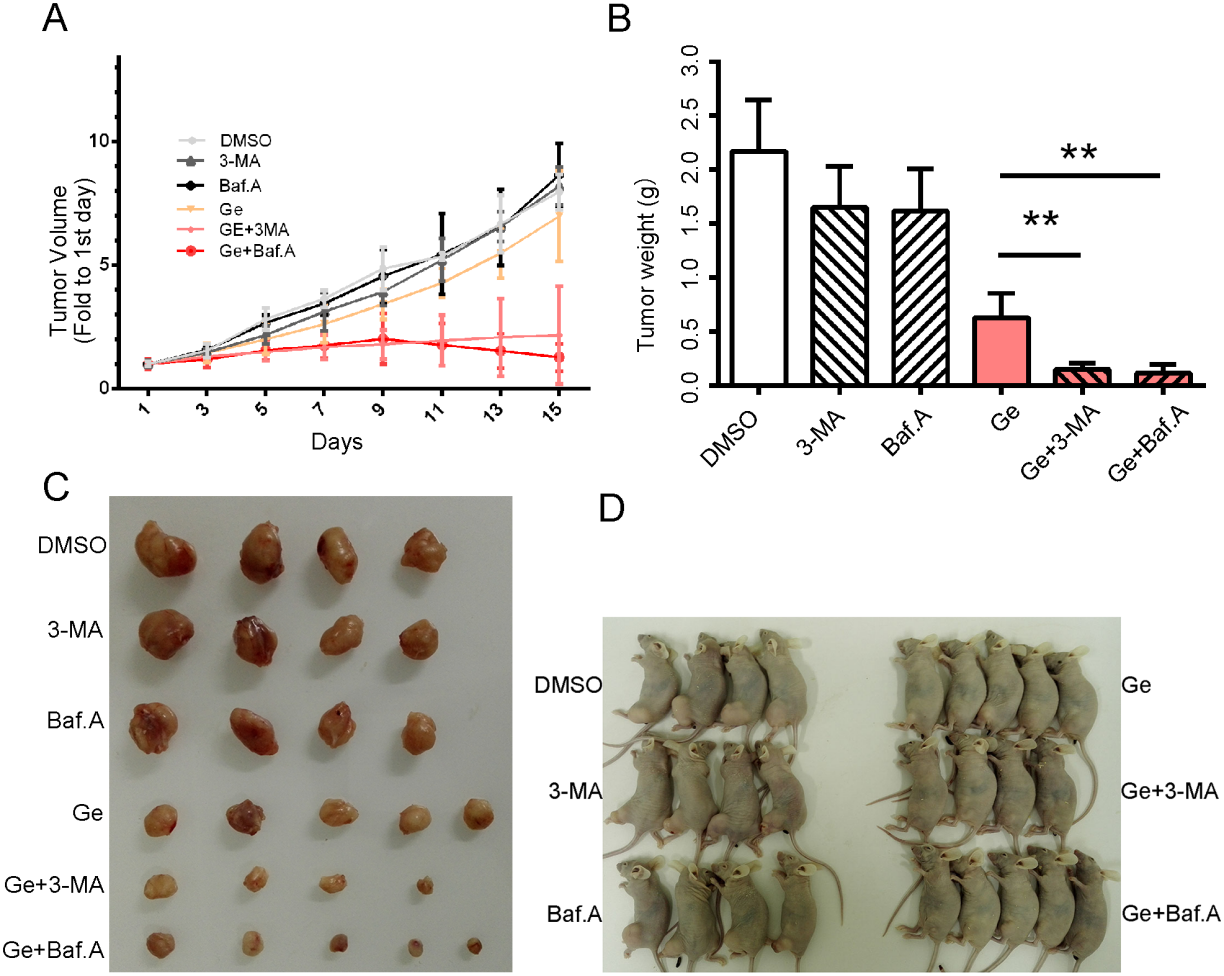
Autophagy inhibitor facilitates the sensitivity of Ge in TNBC xenografts *in vivo*. MDA-MB-468 xenograft tumor was established and treated as follows: DMSO, 3-MA (1.0 mg/kg), Baf.A (1.0 mg/kg), Ge (100 mg/kg), Ge (100 mg/kg) +3-MA (1.0 mg/kg) and Ge (100 mg/kg) +Baf.A (1.0 mg/kg). 3-MA and Baf.A were injected intratumorally, Ge was administered via oral gavage. Tumor growth curves **(A)**, tumor weight **(B)**, tumor image **(C)**, and nude mouse image **(D)** with different treatment were detected. The results are shown as means ± SD; *p < 0.05.

To confirm the facilitation of autophagy inhibition on Ge *in vivo*, xenografts derived from MDA-MB-468 were constructed. As expected, tumors administrated with combination of autophagy inhibitor and Ge grew slower than those with monotherapy (Fig 3A). Accordingly, the weight and size of separated tumors were smaller in combined therapy groups than those in monotherapy groups (Fig 3B, 3C and 3D), indicating combined treatment of autophagy inhibitor with Ge were substantially effectively than that of Ge treatment alone, along with the results in vitro. Taken together, the date provides further understanding with the cytotoxic effect of autophagy inhibitor on Ge against TNBC cells in vivo.

### Autophagy inhibitor facilitates Ge induced G0/G1 arrest and DNA damage repair pathway activation in TNBC cells

**Fig 4:**
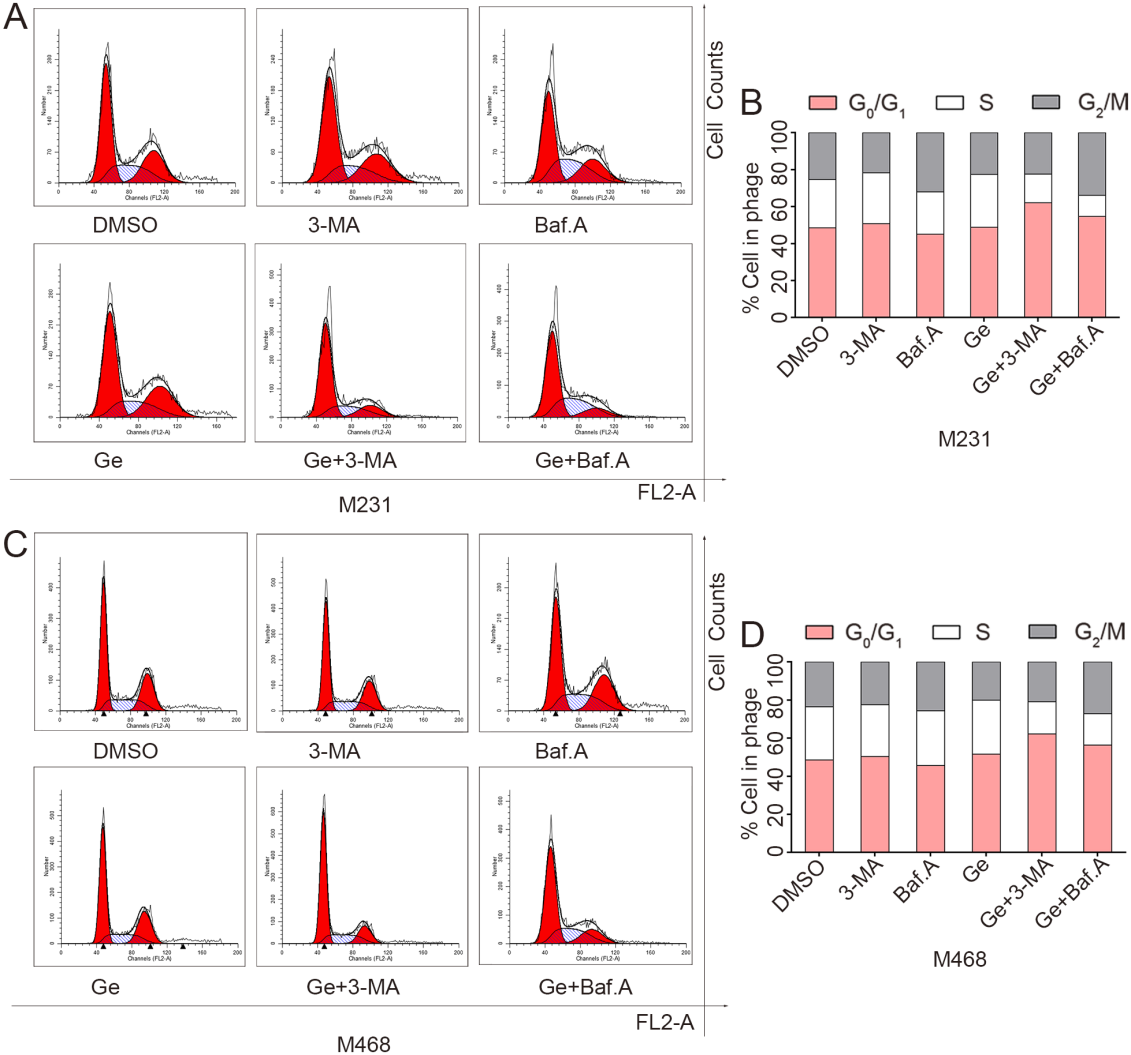
Autophagy inhibitor facilitates Ge induced G0/G1 arrest in TNBC cells. Cells were treated with DMSO, 3-MA, Baf.A, Ge, Ge+3-MA and Ge+Baf.A for 48 hours. The cycle distributions of MDA-MB-231 and MDA-MB-468 cells were analyzed.

**Fig 5:**
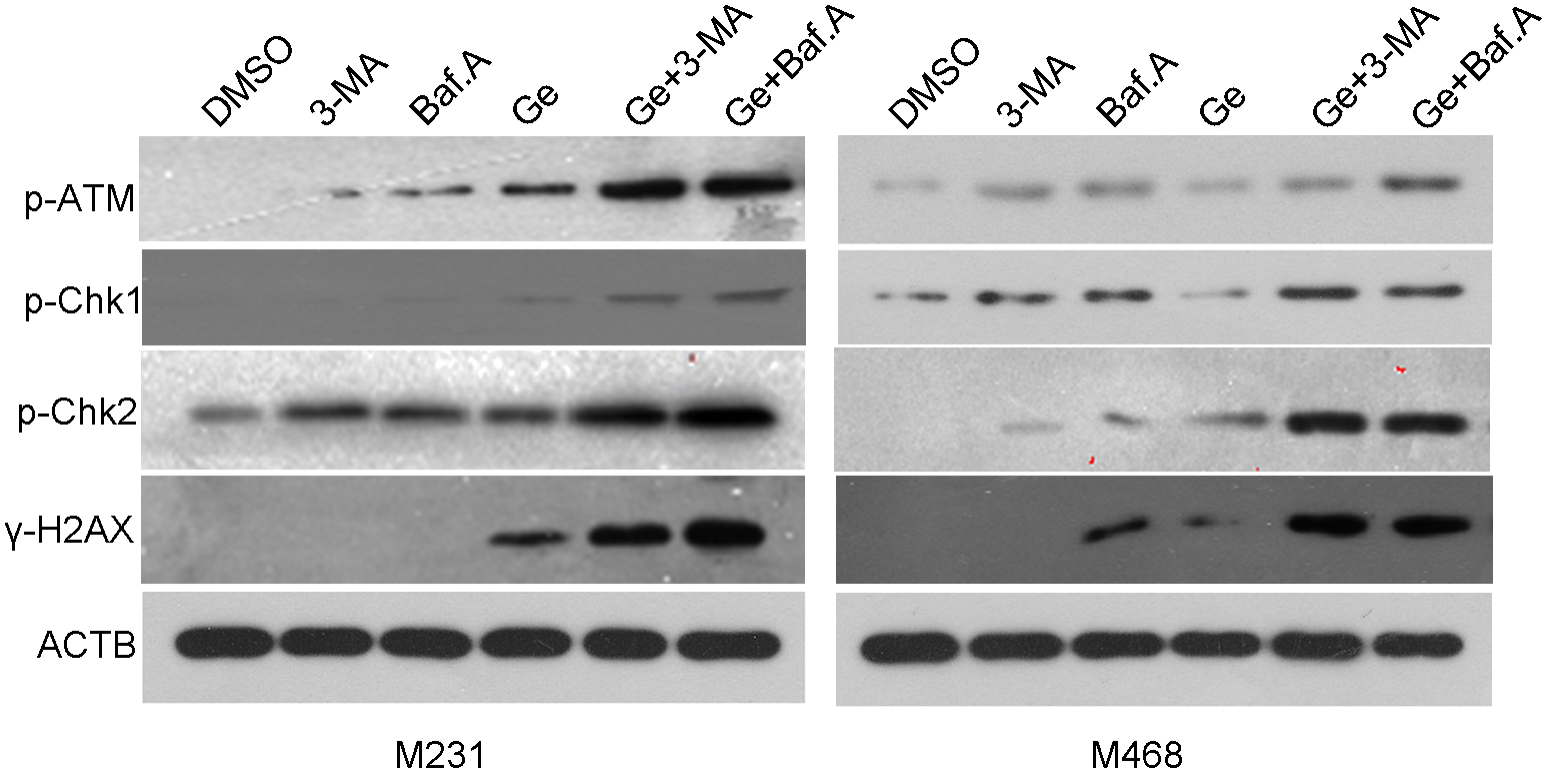
Autophagy inhibitor facilitates DNA damage in TNBC cells. Cells were treated with DMSO, 3-MA, Baf.A, Ge, Ge+3-MA and Ge+Baf.A for 48 hours. Phospho-ATM, Phospho-Chk1, Phospho-Chk2, γ-H2AX, and ACTB of MDA-MB-231 and MDA-MB-468 cells were analyzed by western blot.

Cell-cycle distribution was analyzed to verify the synergistic effect of autophagy inhibitor and Ge by flow cytometry. MDA-MB-231 and MDA-MB-468 were exposed with DMSO, 3-MA, Baf.A, Ge, Ge+3-MA and Ge+Baf.A for 48 hours, respectively. As shown in Fig 4, combined treatment (Ge+3-MA, or Ge+Baf.A) increased the population at the G0/G1 phase and decreased the population at the G2/M and S phase in all tested cells compared to Ge alone. These results revealed that the combined treatment arrested MDA-MB-231 (Fig 4A and 4B) and MDA-MB-468 (Fig 4C and 4D) cell cycle at the G0/G1 phase. ATM recruited to the damaged site when DNA was damaged. The activated of ATM phosphorylated histone H2AX (yielding γ-H2AX) and transmited the DNA damage related signaling molecules, including phosphorylated Chk1 and Chk2 [27]. As shown in Fig 5, western blot showed that DNA damage related molecular such as Phospho-Chk1, Phospho-Chk2, Phospho-ATM, Phospho-Histone H2AX were overexpressed in the combined groups.

### Autophagy inhibitor facilitates Ge induced cell death via mitochondrial apoptosis pathway

**Fig 6:**
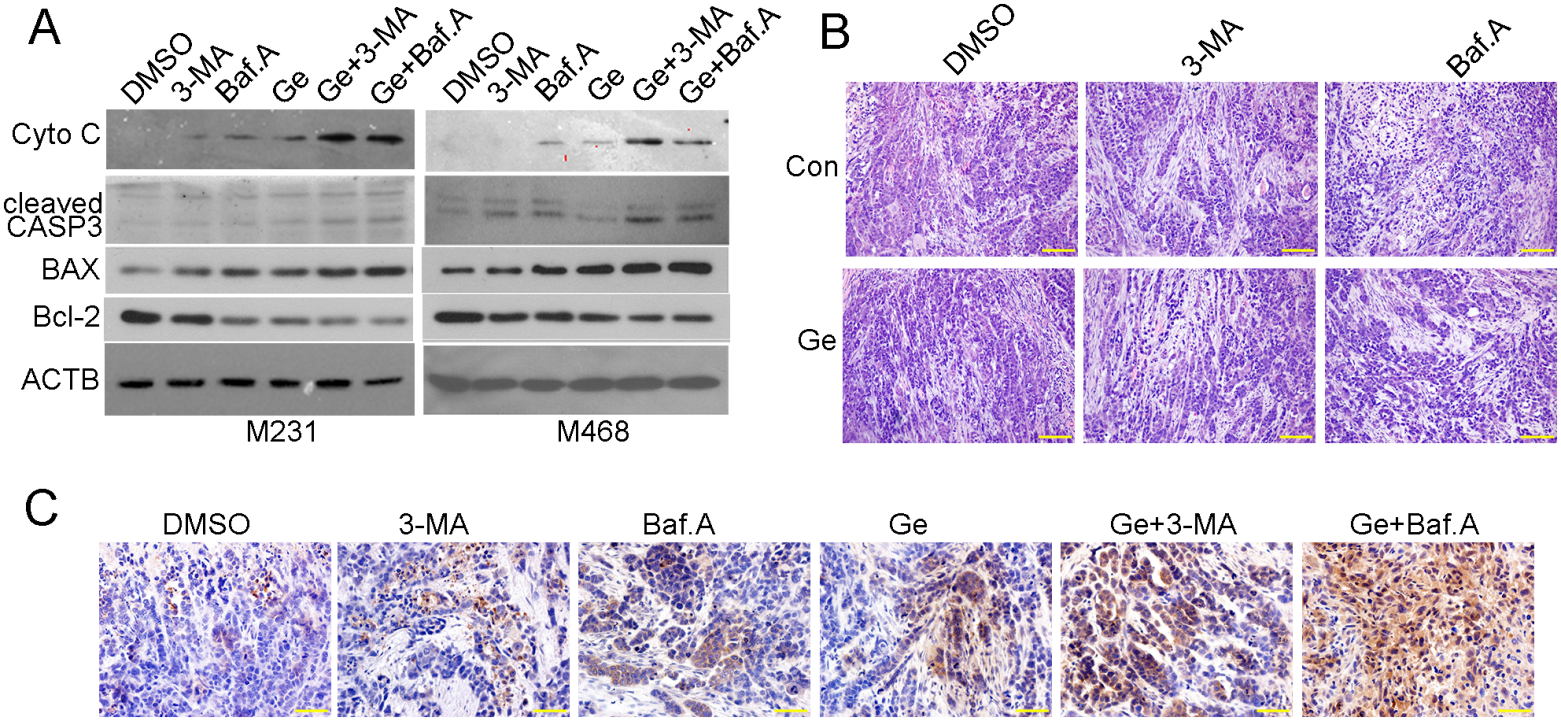
Autophagy inhibitor facilitates Ge induced cell death via mitochondrial apoptosis pathway. The above cells were treated with DMSO, 3-MA, Baf.A, Ge, Ge+3-MA and Ge+Baf.A for 48 hours and were subjected to western blot using following antibodies, Cytochrome C (Cyto C), cleaved CASP3, BAX, Bcl-2 and ACTB **(A)**. Hematoxylin-eosin staining **(B)** and immunohistochemical assays (anti-cleaved CASP 3) **(C)** were performed in MDA-MB-468 xenograft.

In order to determine whether tumor cells treated with above drugs underwent cell death, we performed western blot and immunohistochemical assay to detect changes of protein expression in apoptosis related pathway. More positive results were showed by immunohistochemical assay when treated with combined drug other than treated with Ge alone in MDA-MB-468 xenograft (Fig 6B and 6C). To further confirm the underlying apoptosis signal pathway, we detected apoptosis related gene expression in MDA-MB-231 and MDA-MB-468 cells. As shown in Fig 6A, combined drugs treatment led to an increase in expression of cleaved CASP 3, indicating it might induce cell death in a caspase-dependent manner. Notably, Cytochrome C and the rate of BAX/Bcl-2 were elevated in response to combined drugs treatment, all these genes were vital markers involved in mitochondrial apoptosis pathway, indicating that mitochondrial apoptosis was activated and played a prominent role in the process that autophagy inhibitor facilitates Ge induced cell death.

## Discussion

Ge, a small-molecule TKI against EGFR, inhibits cancer growth mainly through targets the adenosine triphosphate binding sites in the cytoplasmic domain of EGFR [28–30]. Although Ge had been widely used in EGFR^+^ NSCLC patients, unfortunately, it demonstrated little effectiveness in treating breast cancer [31, 32]. The interactions of EGFR downstream pathways, including PI3K/Akt and ERK1/2 pathway, led to continued activation of EGFR downstream molecular and insensitivity toward TKI [33]. Intrinsic resistance to Ge remains a main obstacle in the therapy of TNBC. In this research, we proposed autophagy inhibitor could facilitate the sensitivity of Ge in EGFR^+^ TNBC cells.

Insufficient blood supply and nutritional deprivation lead to cell death in established tumors growth. Autophagy is a self-degradation process that promote tumor cell survival under stress through degrading and recycling intracellular constituents to provide cells with energy [34, 35]. Autophagy also contributes to the survival excellence of cancer cells under therapeutic stress and facilitates their drug resistance in diverse types of cancer. EGFR targeted therapy led to varied autophagic response which plays a protective role and is associated with resistance for established tumors. Evidence reported that small molecule EGFR-TKI targeting EGFR can increase autophagy in head and neck squamous cell carcinoma (HNSCC) cells, ovarian cells, and bladder cancer cells [36–38]. The inhibition of autophagy might be a treatment strategy to overcome drug resistance of TKI in EGFR expression patients [39–43]. In our study, 3-MA (an early stage autophagy inhibitor) as well as Baf.A (a late stage autophagy inhibitor), both enhanced gefitinib-induced cell death. Our data showed that the combined groups with autophagy inhibitor and Ge seemed to cause mitochondrial dysfunction accompanied with Cytochrome C expression, which activated caspase signaling pathways.

Mitochondria, which plays a crucial role in apoptosis signal transduction process, regulates autophagy via multiple mechanisms [44]. In turn, defective autophagy enhances the accumulation of damaged mitochondria, and appeared to induce apoptosis via mitochondrial DNA damage [45–47]. Evidence reported that defective mitochondrial could lead to DNA damage, which then would activate ATM. DNA damage signal was transmitted by ATM to downstream targets including p-Chk1 and p-Chk2 which played an important role in cell cycle regulation [48–51]. When autophagy was blocked, damaged mitochondria would release ROS and induce of G0/G1 cell cycle arrest [26, 52], causing oxidation of DNA and resulting in DNA damage. In this study, we found that as the measure molecular of DNA damage, the expression of γ-H2AX, ATM, p-Chk1 and p-Chk2 were increased when Ge and autophagy inhibitor combined. Our findings confirmed that the synergistic treatment with Ge and autophagy inhibitor results in the G0/G1 cell cycle arrest of tumor cells when DNA is damaged beyond repair [53].

Cytochrome C release and BAX activation with the process of mitochondrial damage, are considered as the key components in mitochondrial apoptosis [54, 55]. The Bcl-2 family proteins include the pro-apoptotic Bcl-2 proteins (e.g., BAX and BAK) and the anti-apoptotic Bcl-2 proteins (e.g., Bcl-2 and Bcl-xL). The pro-apoptotic Bcl-2 proteins play a key role in the regulation of MOMP and apoptosis by combination with the Bcl-2 homology domain-only (BH3-only) subclass [56]. The intrinsic apoptosis pathway, as known as mitochondrial apoptosis, begins with BH3 protein induction or activation, which leads to the inactivation of Bcl-2 and the activation of BAX and BAK. Cytochrome C release and mitochondrial fission were enhanced with the activation of BAX and BAK, which results in the activation of apoptotic protease activating facter-1 (APAF1) into an apoptosome and activates the caspase dependent apoptosis [57, 58]. In our study, the proteins associated with mitochondrial apoptosis, including Cytochrome C, BAX, and cleaved caspase-3 were found in the combined treatment with Ge and autophagy inhibitor. Taken together, our findings indicated the involvement of the mitochondrial apoptosis pathway in the process that autophagy inhibitor enhanced the sensitivity of Ge in TNBC cells.

## Conclusions

Collectively, we have shown that autophagy inhibitor facilitates the cytotoxicity of Ge in TNBC cells. Autophagy inhibitor appears to be useful as a potential candidate for TNBC targeted therapy.

## Supporting information

S1 Dataset The supporting information of CCK8. (XLSX)

S2 Dataset The supporting information of Colony formation assay. (DOCX)

S3 Dataset Dates of tumor growth. (XLSX)

S4 Dataset The original date of cell cycle distribution from the flow cytometric analysis. (DOCX)

S5 Dataset Images of western blot. (DOCX)
