# Supplementary figures and images for: Autophagy inhibitor facilitates gefitinib sensitivity in vitro and in vivo by activating mitochondrial apoptosis in triple negative breast cancer
Source: PLoS One. 2017 May 22;12(5):e0177694. doi: 10.1371/journal.pone.0177694 (PMC5439698; doi:10.1371/journal.pone.0177694)

231 DMSO


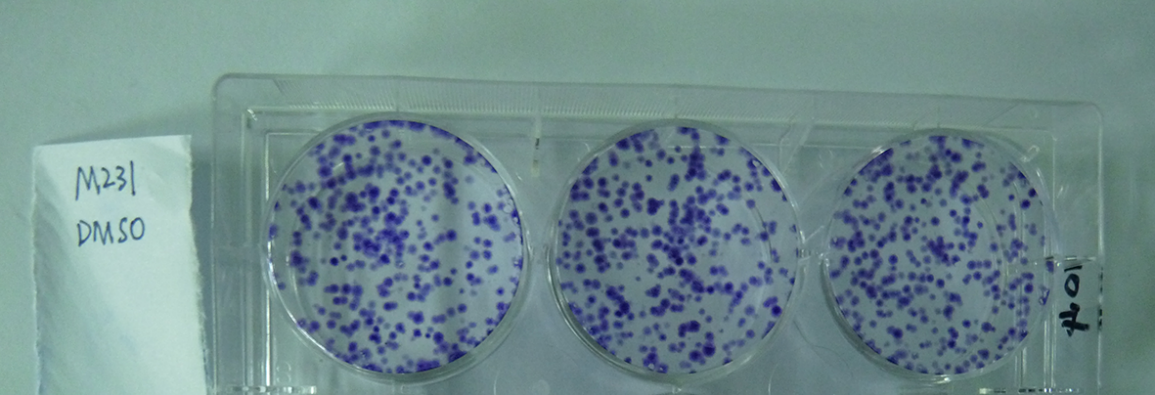


231 3-MA


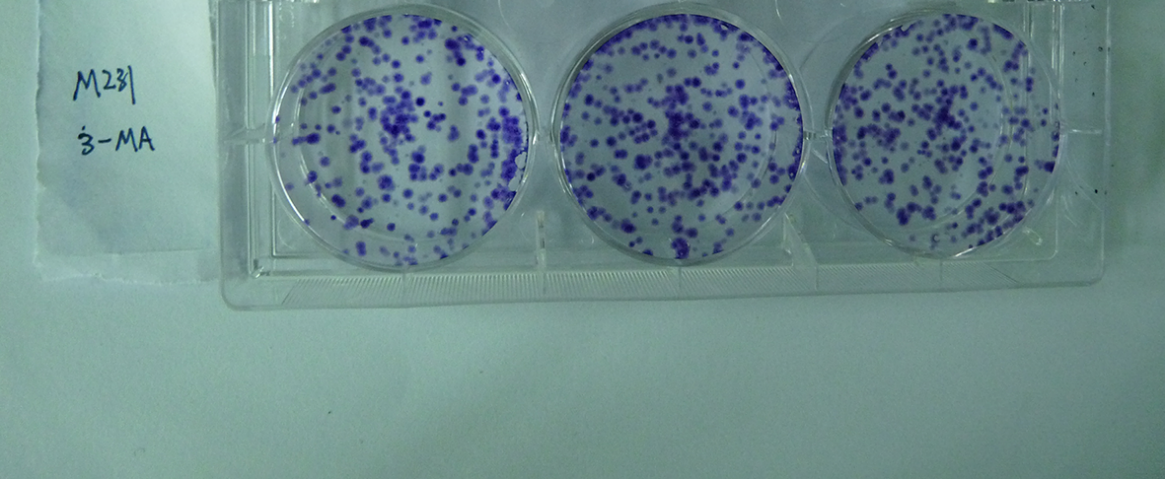


231 Baf.A


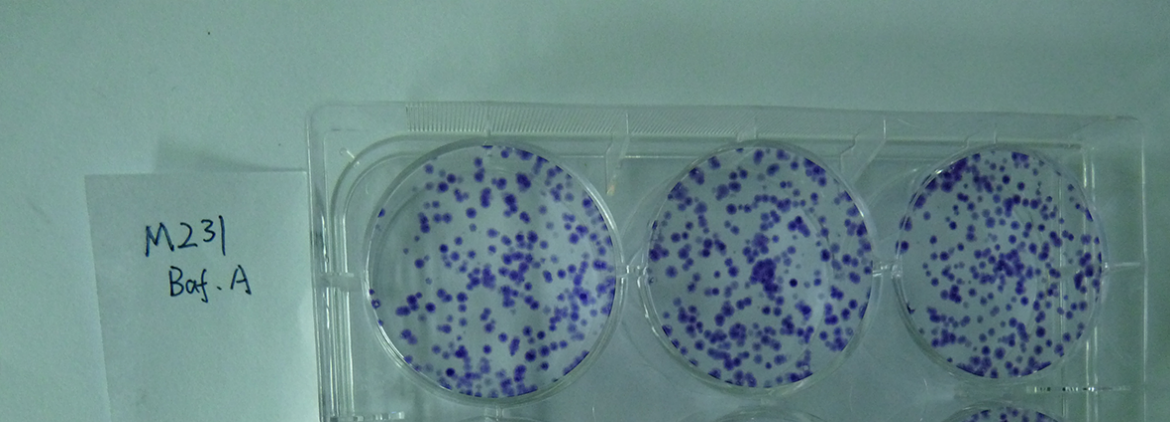


231 Ge


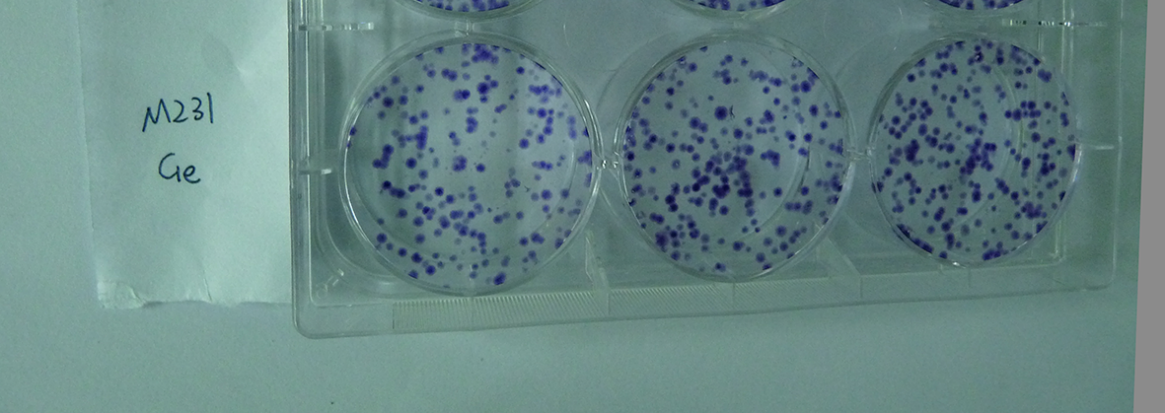


231 Ge+3MA


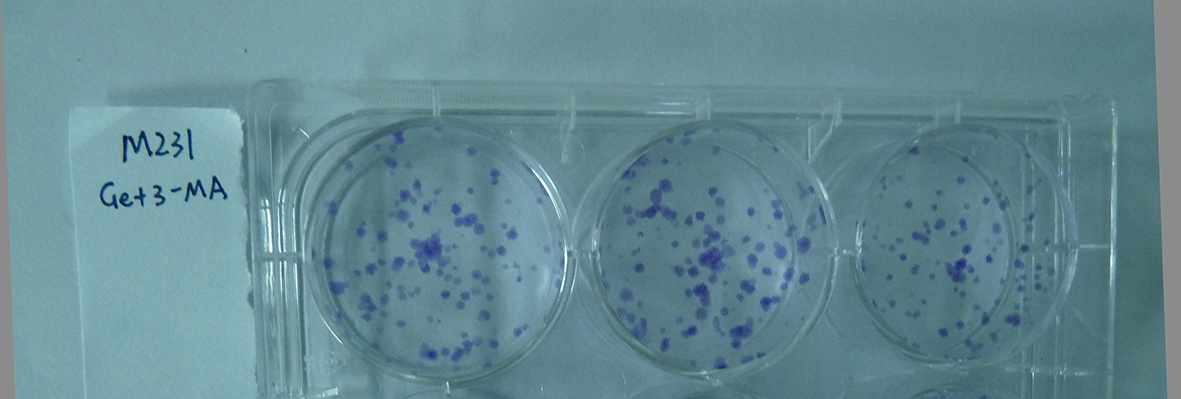


M231 Ge+Baf.A


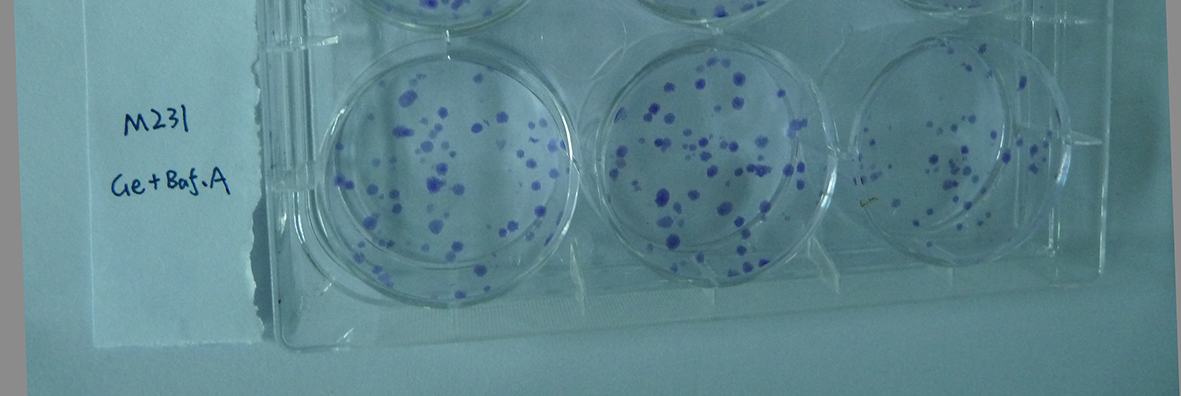


M468 DMSO


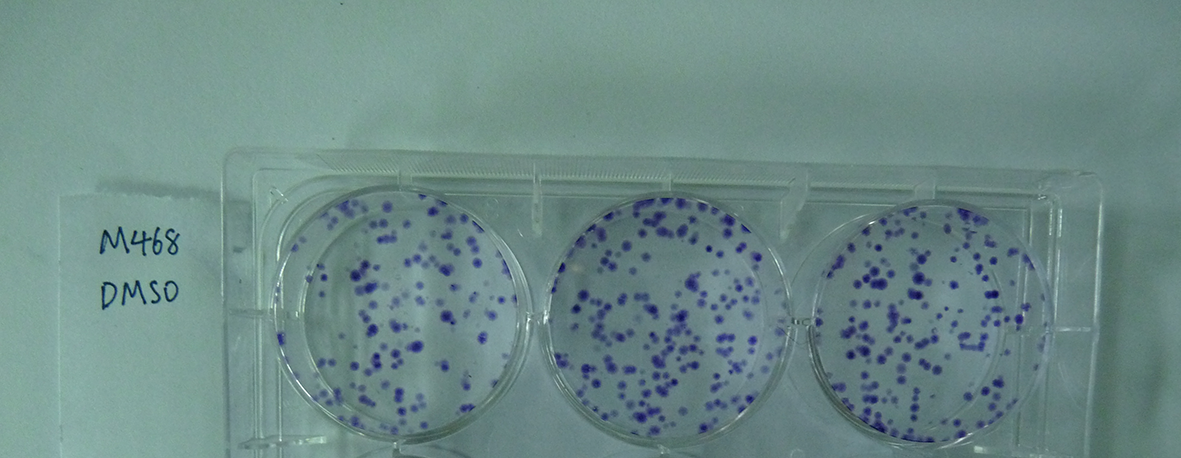


M468 3MA


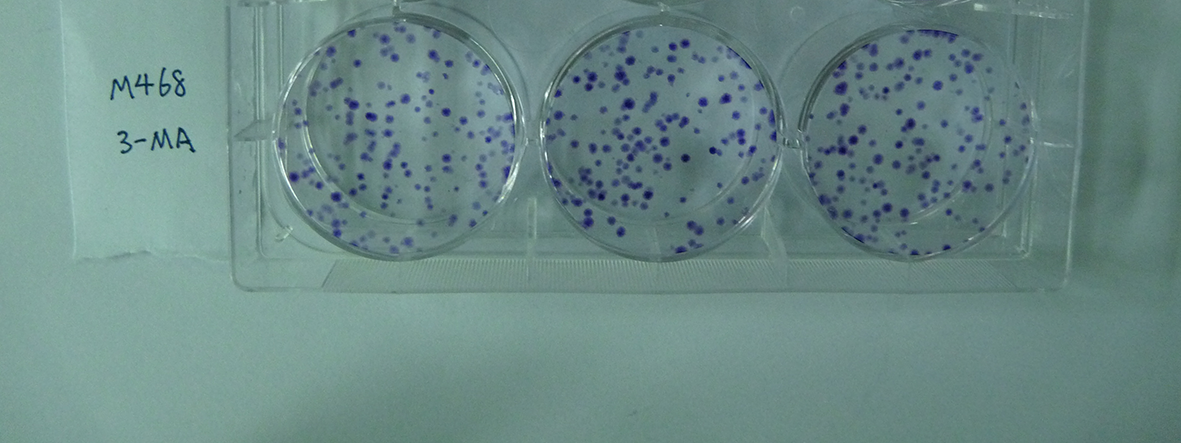


M468 Baf.A


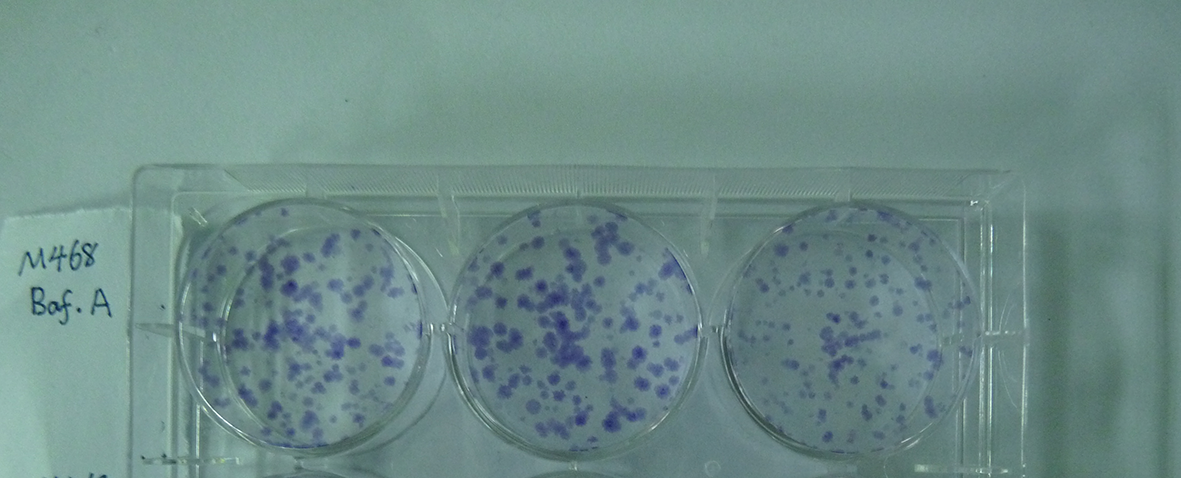


M468 Ge


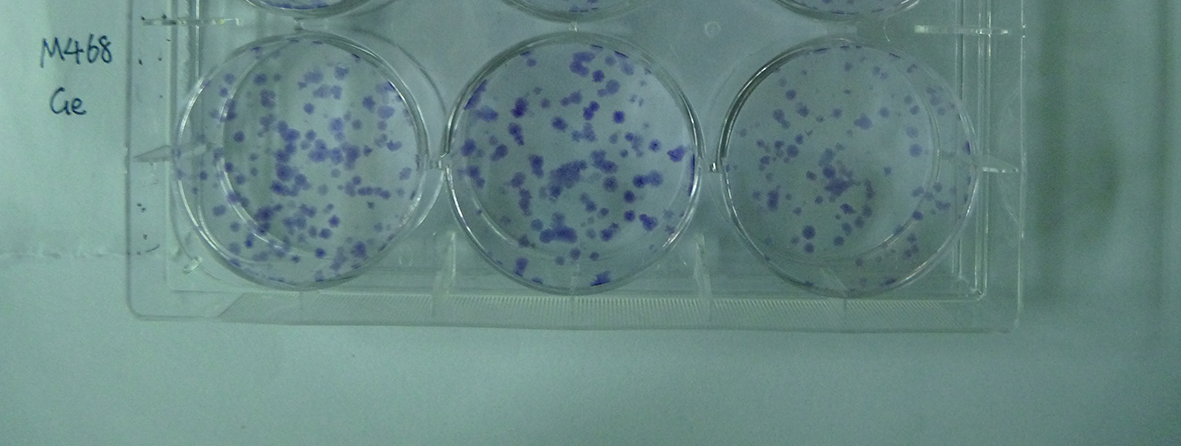


M468 Ge+3MA


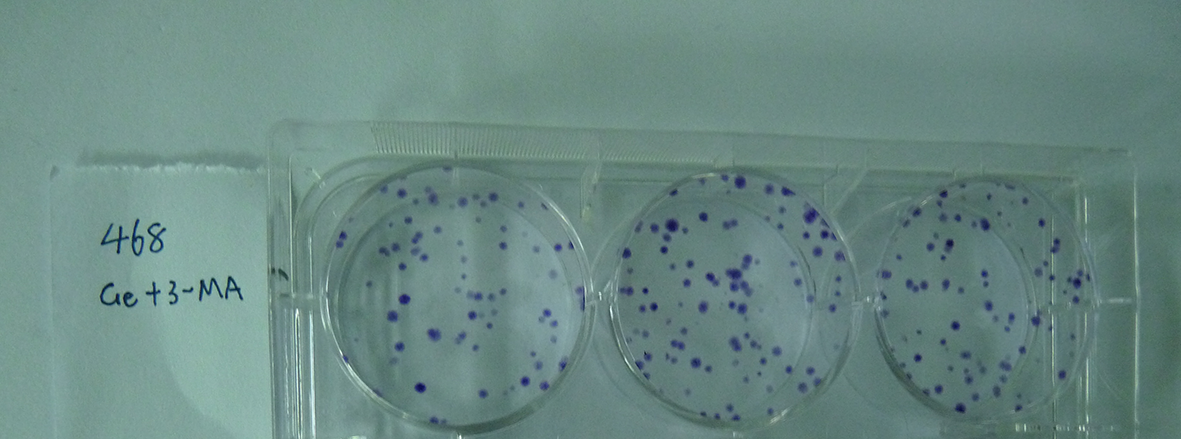


M468 Ge+Baf.A


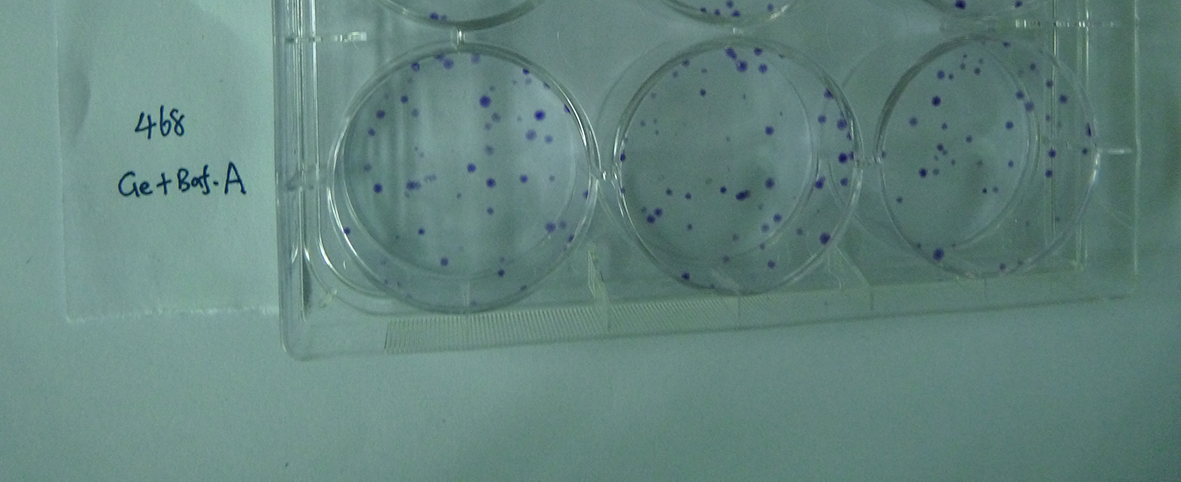

Supplement: S2 Dataset — (DOCX) [file pone.0177694.s002.docx]
