# Supplementary material for: Autophagy inhibitor facilitates gefitinib sensitivity in vitro and in vivo by activating mitochondrial apoptosis in triple negative breast cancer
Source: PLoS One. 2017 May 22;12(5):e0177694. doi: 10.1371/journal.pone.0177694 (PMC5439698; doi:10.1371/journal.pone.0177694)
Supplement: S4 Dataset — (DOCX) [file pone.0177694.s004.docx]

**M-231**


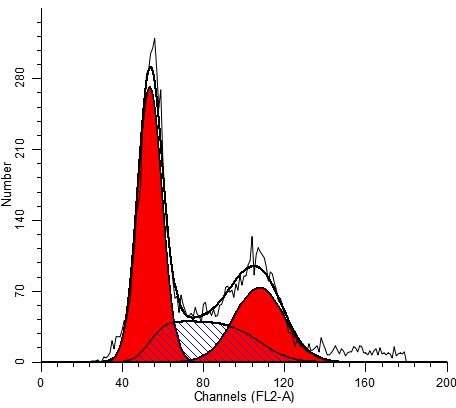
File analyzed: M2310

Date analyzed: 13-Apr-2016

Model: 1nn0n_DSD

Analysis type: Manual analysis

Ploidy Mode: First cycle is diploid

Diploid: 100.00 %

Dip G1: 48.54 % at 53.89

Dip G2: 26.05 % at 107.78

Dip S: 25.40 % G2/G1: 2.00

%CV: 11.42

Total S-Phase: 25.40 %

Total B.A.D.: 0.00 % no debris no aggs

Debris: %

Aggregates: 0.00 %

Modeled events: 8644

All cycle events: 8644

Cycle events per channel: 157

RCS: 3.251


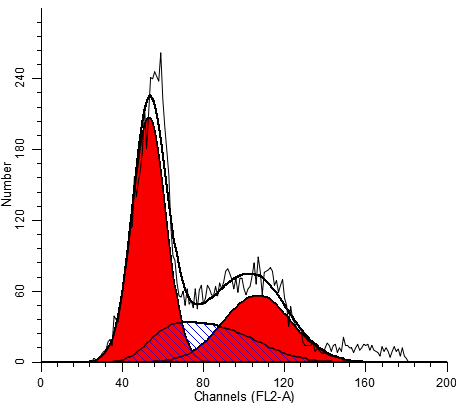
F

ile analyzed: M2313

Date analyzed: 13-Apr-2016

Model: 1nn0n_DSD

Analysis type: Manual analysis

Ploidy Mode: First cycle is diploid

Diploid: 100.00 %

Dip G1: 50.72 % at 53.47

Dip G2: 27.54 % at 106.95

Dip S: 21.75 % G2/G1: 2.00

%CV: 15.61

Total S-Phase: 21.75 %

Total B.A.D.: 0.00 % no debris no aggs

Debris: %

Aggregates: 0.00 %

Modeled events: 8561

All cycle events: 8561

Cycle events per channel: 157

RCS: 3.028


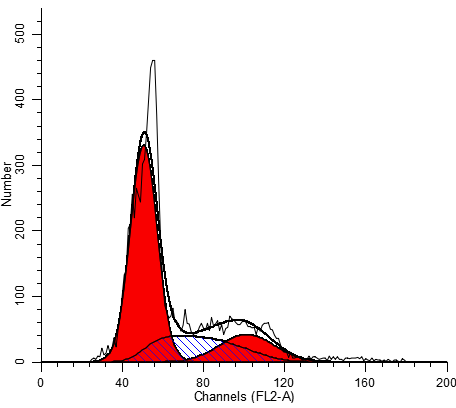


File analyzed: M2313MA+GE

Date analyzed: 13-Apr-2016

Model: 1nn0n_DSD

Analysis type: Manual analysis

Ploidy Mode: First cycle is diploid

Diploid: 100.00 %

Dip G1: 62.02 % at 50.89

Dip G2: 15.51 % at 101.78

Dip S: 22.48 % G2/G1: 2.00

%CV: 13.15

Total S-Phase: 22.48 %

Total B.A.D.: 0.00 % no debris no aggs

Debris: %

Aggregates: 0.00 %

Modeled events: 8964

All cycle events: 8964

Cycle events per channel: 173

RCS: 3.942


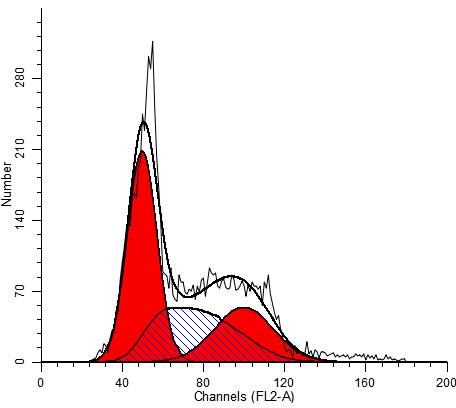


File analyzed: M231BAF

Date analyzed: 13-Apr-2016

Model: 1nn0n_DSD

Analysis type: Manual analysis

Ploidy Mode: First cycle is diploid

Diploid: 100.00 %

Dip G1: 45.04 % at 50.11

Dip G2: 22.90 % at 100.21

Dip S: 32.06 % G2/G1: 2.00

%CV: 14.66

Total S-Phase: 32.06 %

Total B.A.D.: 0.00 % no debris no aggs

Debris: %

Aggregates: 0.00 %

Modeled events: 8542

All cycle events: 8542

Cycle events per channel: 167

RCS: 3.111


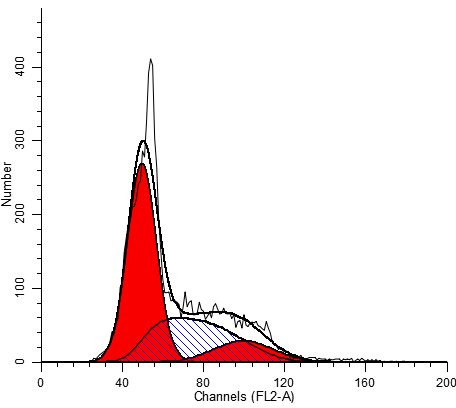
File analyzed: M231BAF+GE

Date analyzed: 13-Apr-2016

Model: 1nn0n_DSD

Analysis type: Manual analysis

Ploidy Mode: First cycle is diploid

Diploid: 100.00 %

Dip G1: 54.63 % at 49.97

Dip G2: 11.48 % at 99.94

Dip S: 33.89 % G2/G1: 2.00

%CV: 14.41

Total S-Phase: 33.89 %

Total B.A.D.: 0.00 % no debris no aggs

Debris: %

Aggregates: 0.00 %

Modeled events: 8903

All cycle events: 8903

Cycle events per channel: 175

RCS: 2.538


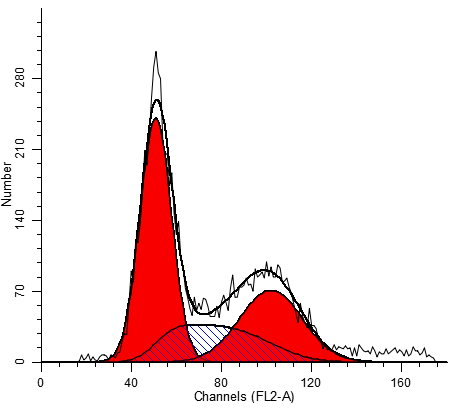


File analyzed: M231GE

Date analyzed: 22-Apr-2016

Model: 1nn0n_DSD

Analysis type: Manual analysis

Ploidy Mode: First cycle is diploid

Diploid: 100.00 %

Dip G1: 48.85 % at 51.11

Dip G2: 28.59 % at 102.21

Dip S: 22.56 % G2/G1: 2.00

%CV: 13.62

Total S-Phase: 22.56 %

Total B.A.D.: 0.00 % no debris no aggs

Debris: %

Aggregates: 0.00 %

Modeled events: 8595

All cycle events: 8595

Cycle events per channel: 165

RCS: 3.196

**M-468**


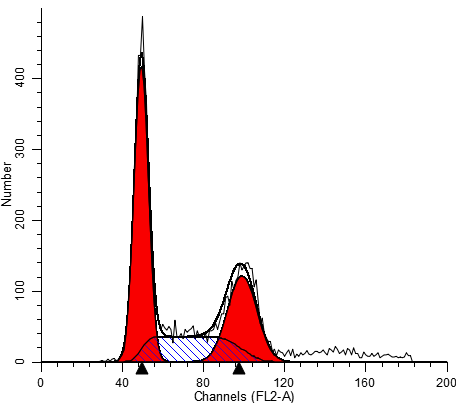
File analyzed: M4680

Date analyzed: 14-Apr-2016

Model: 1nn0n_DSD

Analysis type: Manual analysis

Ploidy Mode: First cycle is diploid

Diploid: 100.00 %

Dip G1: 48.57 % at 49.65

Dip G2: 27.89 % at 99.30

Dip S: 23.54 % G2/G1: 2.00

%CV: 7.13

Total S-Phase: 23.54 %

Total B.A.D.: 0.00 % no debris no aggs

Debris: %

Aggregates: 0.00 %

Modeled events: 7681

All cycle events: 7681

Cycle events per channel: 152

RCS: 4.760


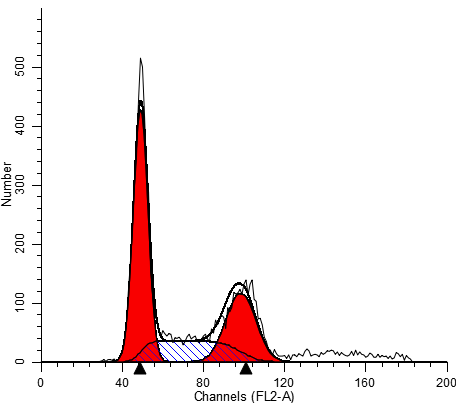
File analyzed: M4683MA

Date analyzed: 14-Apr-2016

Model: 1nn0n_DSD

Analysis type: Manual analysis

Ploidy Mode: First cycle is diploid

Diploid: 100.00 %

Dip G1: 50.41 % at 49.31

Dip G2: 27.16 % at 98.63

Dip S: 22.43 % G2/G1: 2.00

%CV: 7.36

Total S-Phase: 22.43 %

Total B.A.D.: 0.00 % no debris no aggs

Debris: %

Aggregates: 0.00 %

Modeled events: 7748

All cycle events: 7748

Cycle events per channel: 154

RCS: 5.264


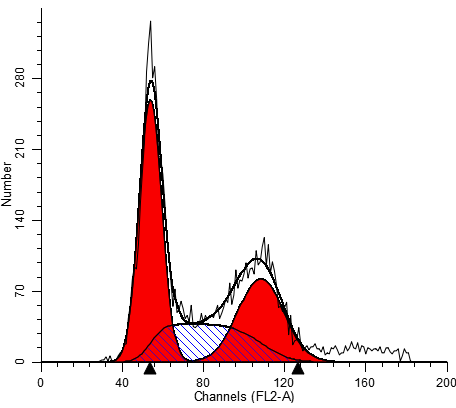


File analyzed: M468B

Date analyzed: 14-Apr-2016

Model: 1nn0n_DSD

Analysis type: Manual analysis

Ploidy Mode: First cycle is diploid

Diploid: 100.00 %

Dip G1: 45.54 % at 54.26

Dip G2: 28.76 % at 108.51

Dip S: 25.70 % G2/G1: 2.00

%CV: 10.32

Total S-Phase: 25.70 %

Total B.A.D.: 0.00 % no debris no aggs

Debris: %

Aggregates: 0.00 %

Modeled events: 8008

All cycle events: 8008

Cycle events per channel: 145

RCS: 3.621


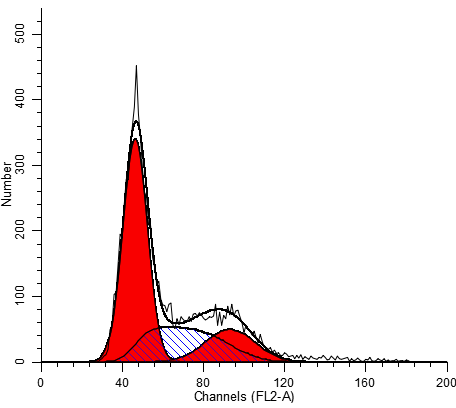


File analyzed: M468B+G

Date analyzed: 14-Apr-2016

Model: 1nn0n_DSD

Analysis type: Manual analysis

Ploidy Mode: First cycle is diploid

Diploid: 100.00 %

Dip G1: 56.36 % at 46.64

Dip G2: 16.43 % at 93.28

Dip S: 27.22 % G2/G1: 2.00

%CV: 13.01

Total S-Phase: 27.22 %

Total B.A.D.: 0.00 % no debris no aggs

Debris: %

Aggregates: 0.00 %

Modeled events: 9195

All cycle events: 9195

Cycle events per channel: 193

RCS: 2.300


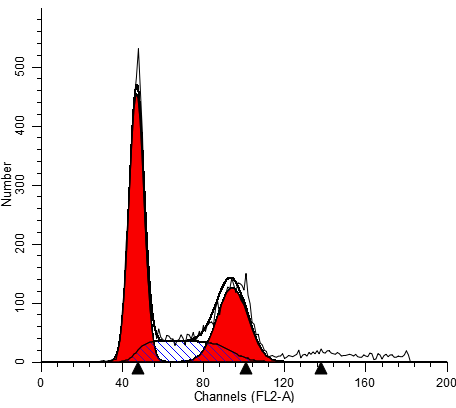


File analyzed: M468GE

Date analyzed: 14-Apr-2016

Model: 1nn0n_DSD

Analysis type: Manual analysis

Ploidy Mode: First cycle is diploid

Diploid: 100.00 %

Dip G1: 51.62 % at 47.29

Dip G2: 28.35 % at 94.58

Dip S: 20.04 % G2/G1: 2.00

%CV: 7.86

Total S-Phase: 20.04 %

Total B.A.D.: 0.00 % no debris no aggs

Debris: %

Aggregates: 0.00 %

Modeled events: 8241

All cycle events: 8241

Cycle events per channel: 171

RCS: 5.165


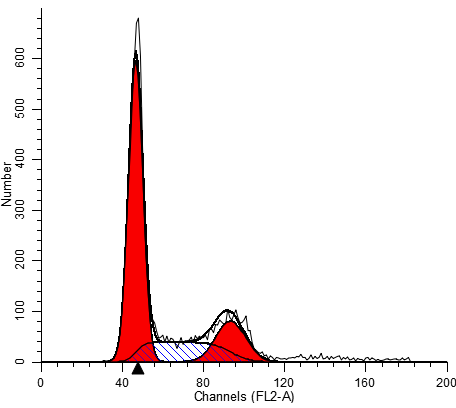


File analyzed: M468GE+3MA

Date analyzed: 14-Apr-2016

Model: 1nn0n_DSD

Analysis type: Manual analysis

Ploidy Mode: First cycle is diploid

Diploid: 100.00 %

Dip G1: 62.21 % at 46.83

Dip G2: 16.81 % at 93.65

Dip S: 20.98 % G2/G1: 2.00

%CV: 7.70

Total S-Phase: 20.98 %

Total B.A.D.: 0.00 % no debris no aggs

Debris: %

Aggregates: 0.00 %

Modeled events: 8683

All cycle events: 8683

Cycle events per channel: 182

RCS: 3.549
