# Supplementary material for: Autophagy inhibitor facilitates gefitinib sensitivity in vitro and in vivo by activating mitochondrial apoptosis in triple negative breast cancer
Source: PLoS One. 2017 May 22;12(5):e0177694. doi: 10.1371/journal.pone.0177694 (PMC5439698; doi:10.1371/journal.pone.0177694)

M231 P-ATM


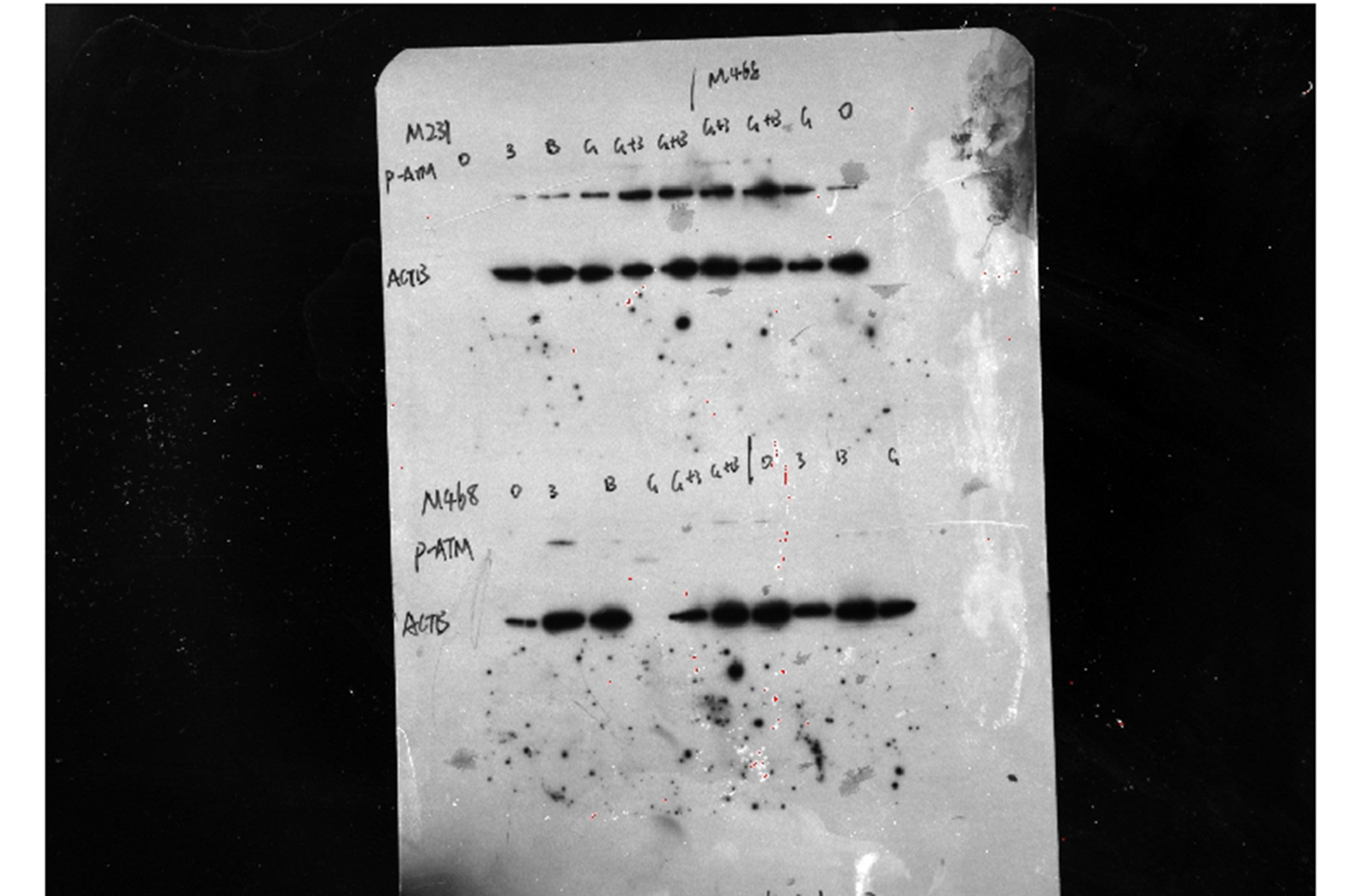


M231 P-Chk1


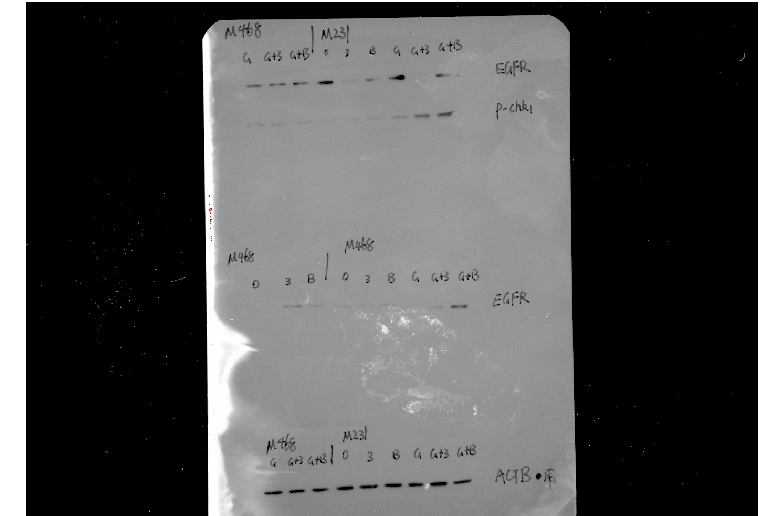


M231 P-Chk2


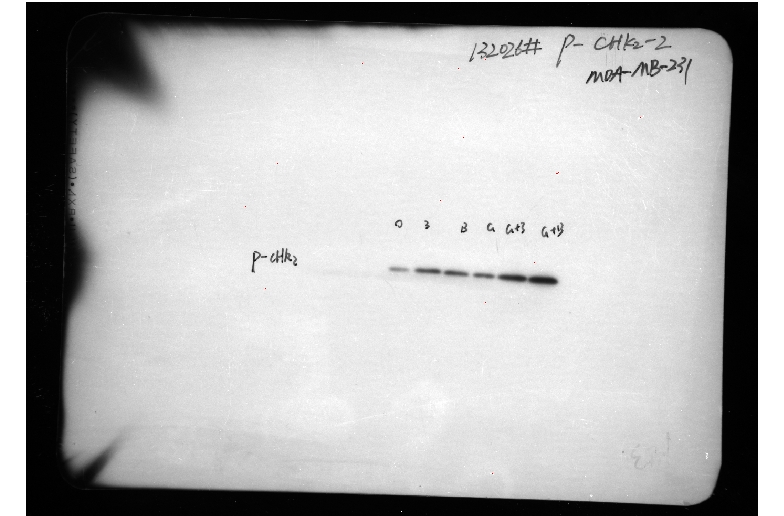


M231 H2AX


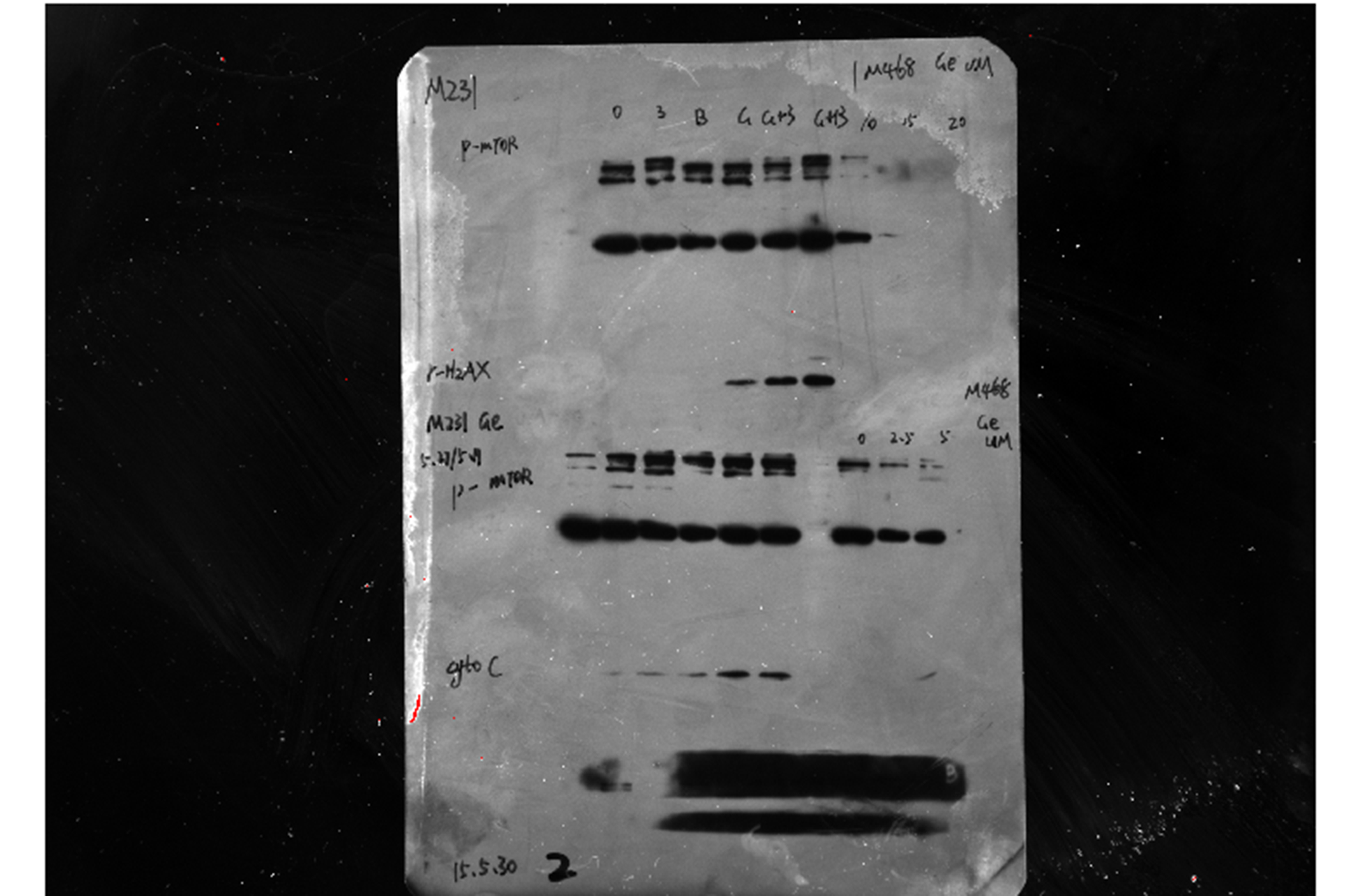


231 ACTB


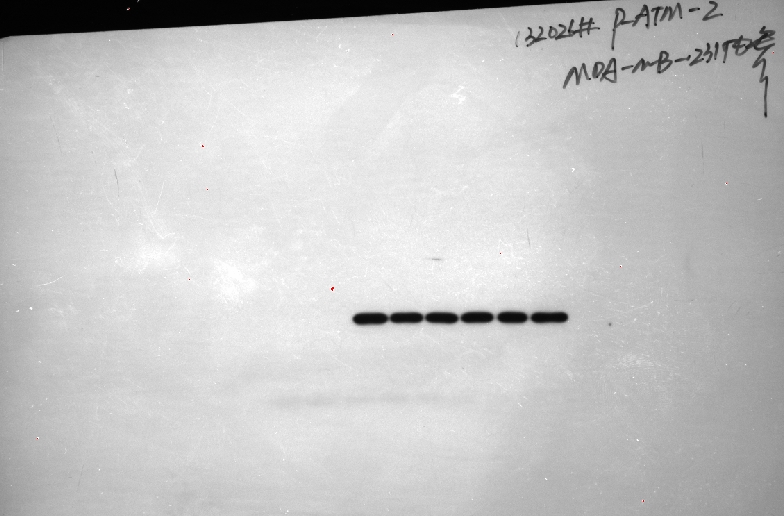


M468 P-ATM


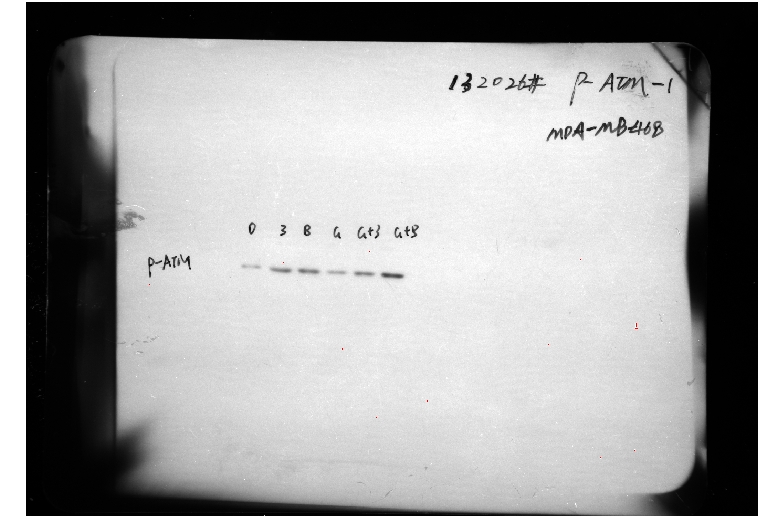


M468 P-Chk1


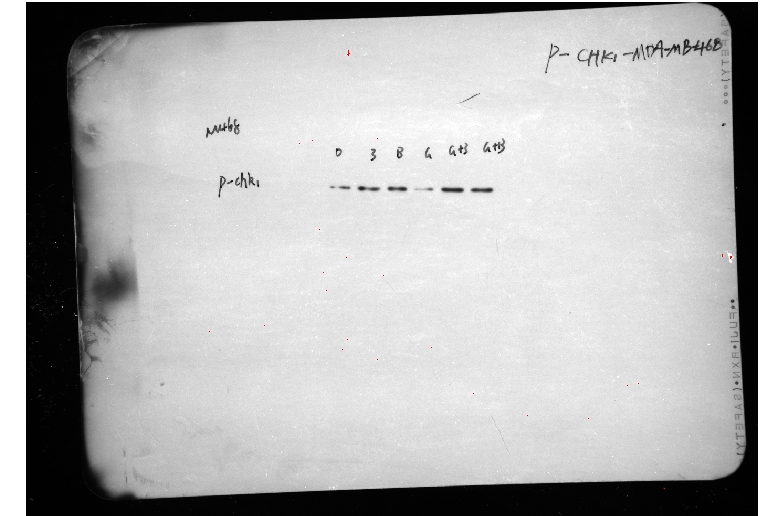


M468 P-Chk2


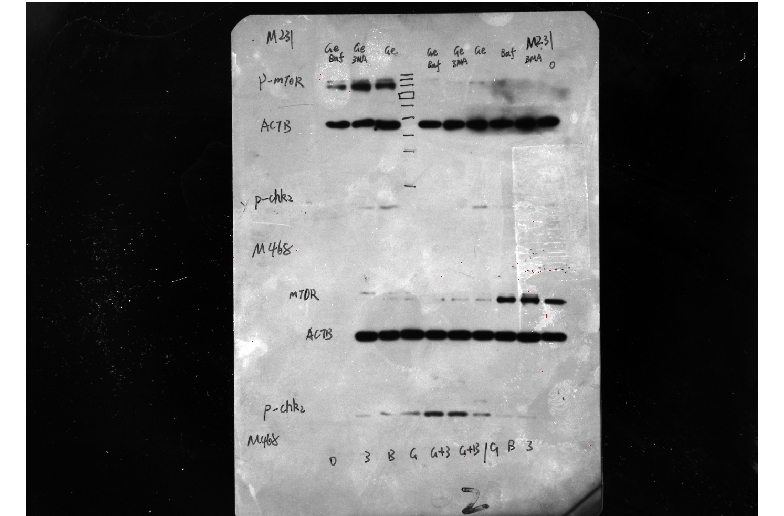


M468 H2AX


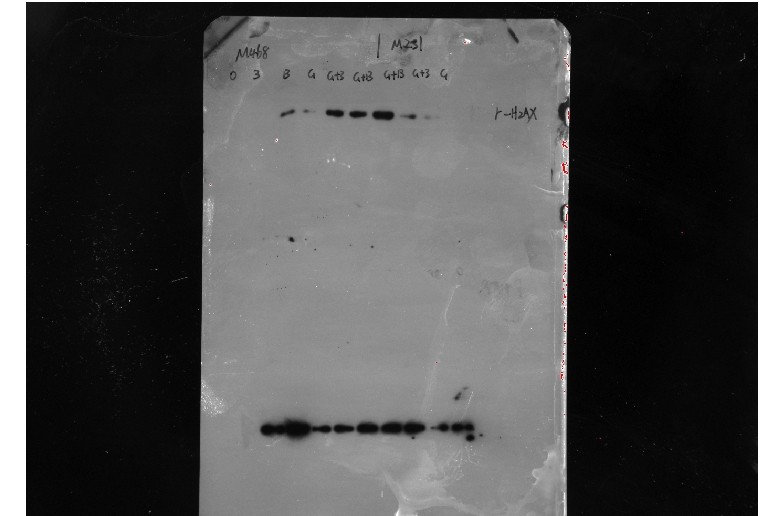


M468 ACTB


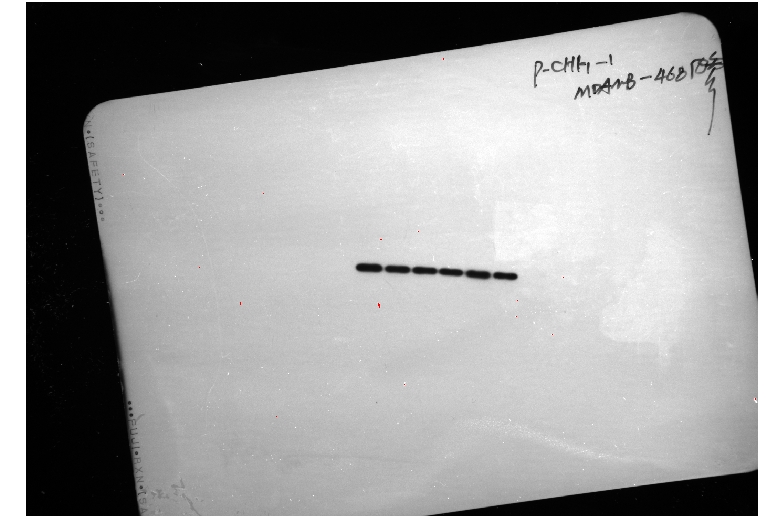


Fig 5

231 Cyto C


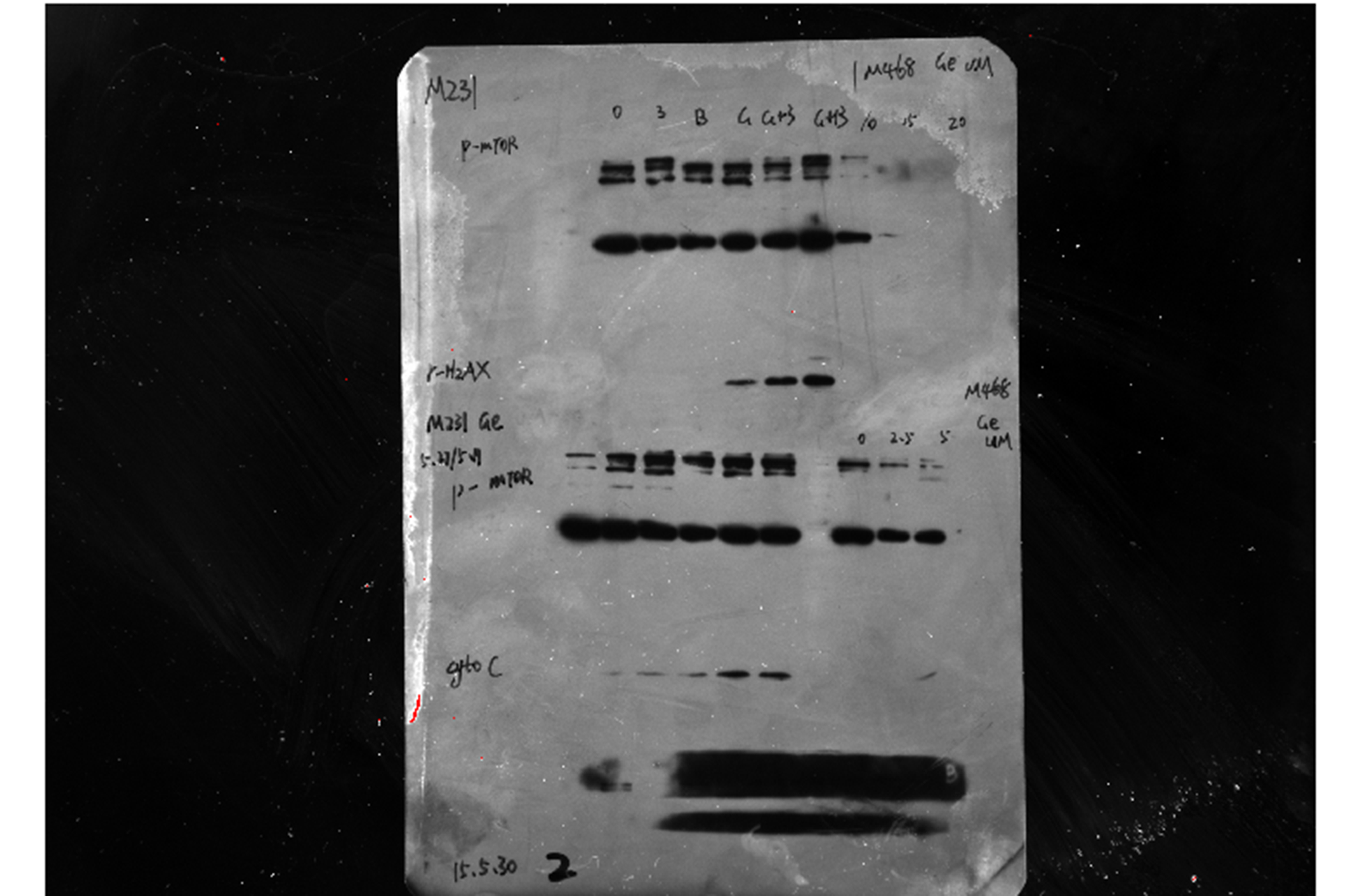


231 Cleaved caspase 3


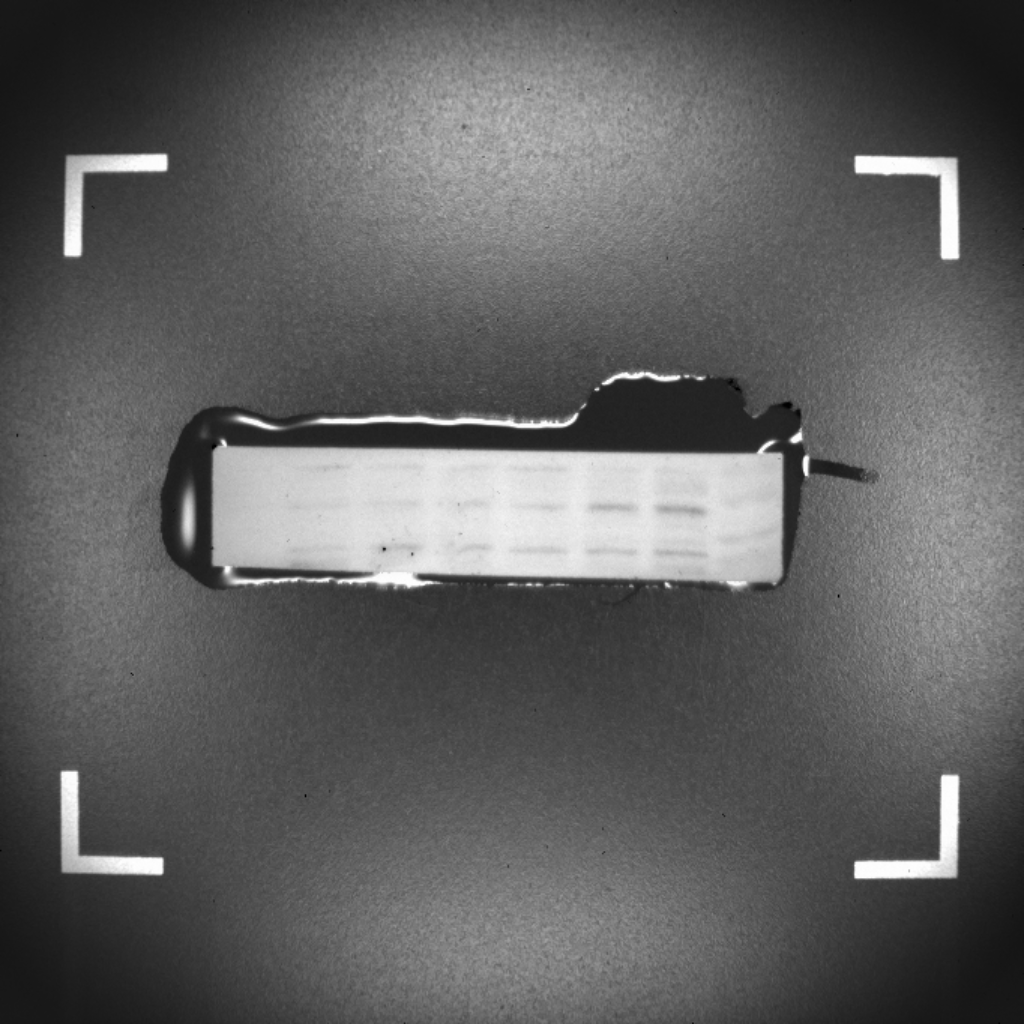


231 BAX


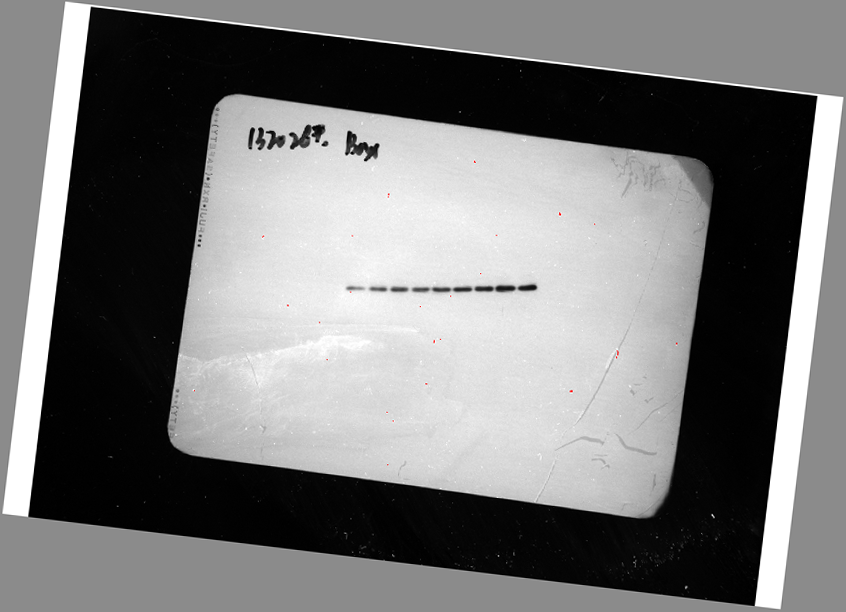


231 Bcl-2


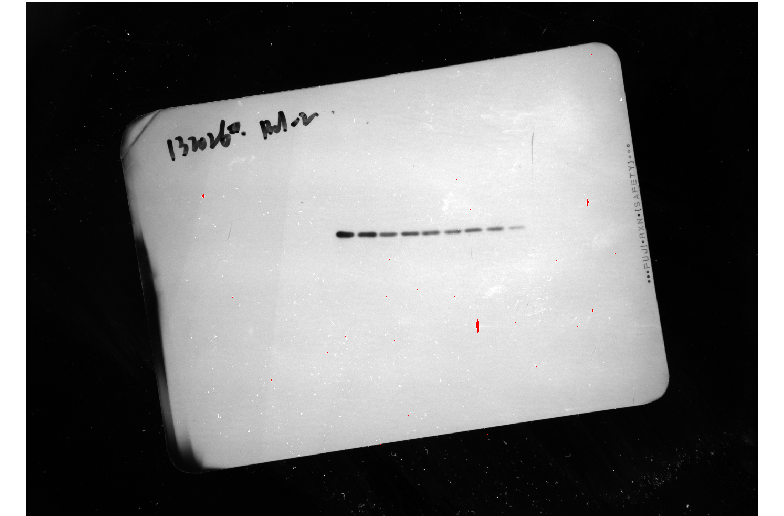


231 ACTB


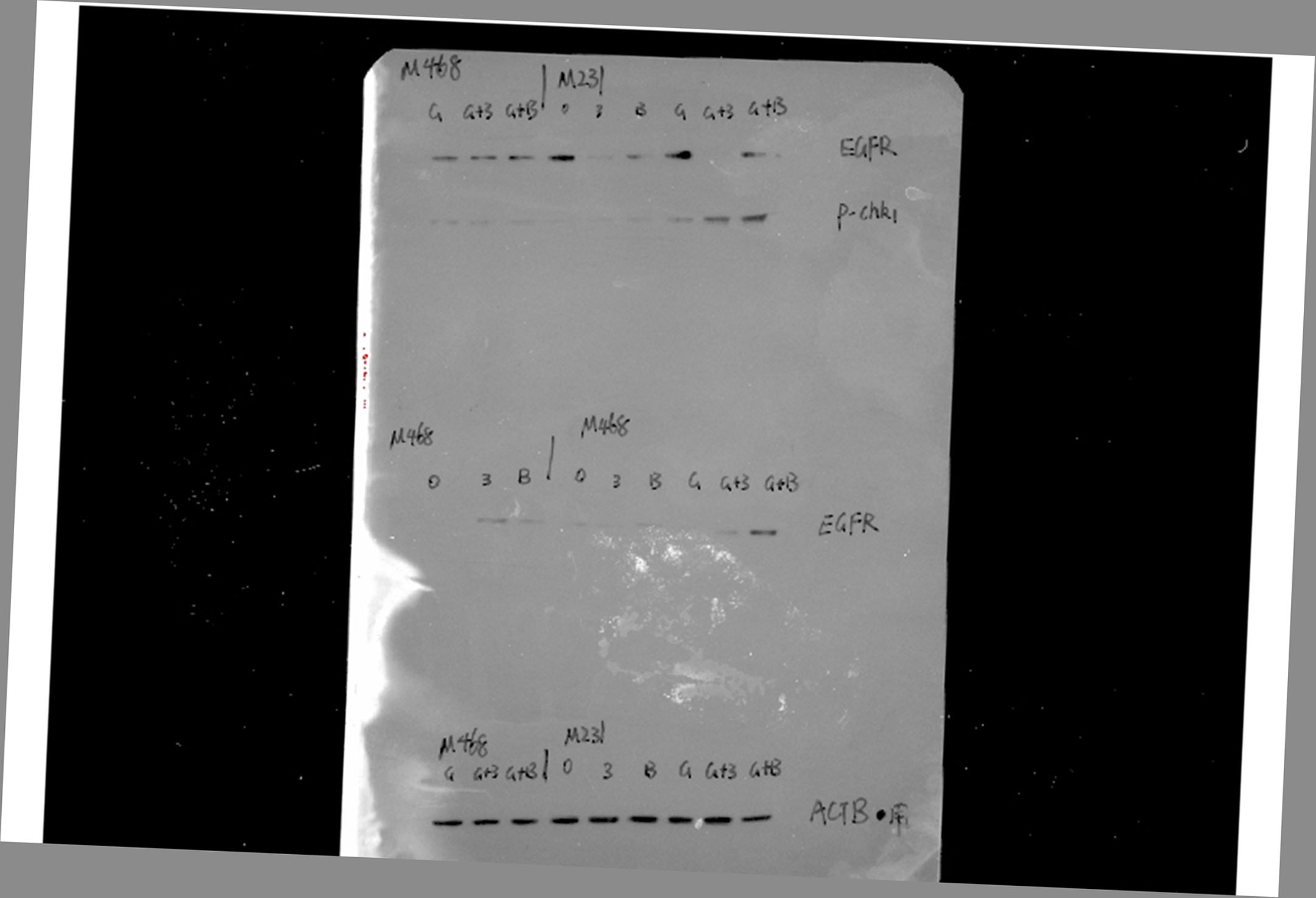


468 Cyto C


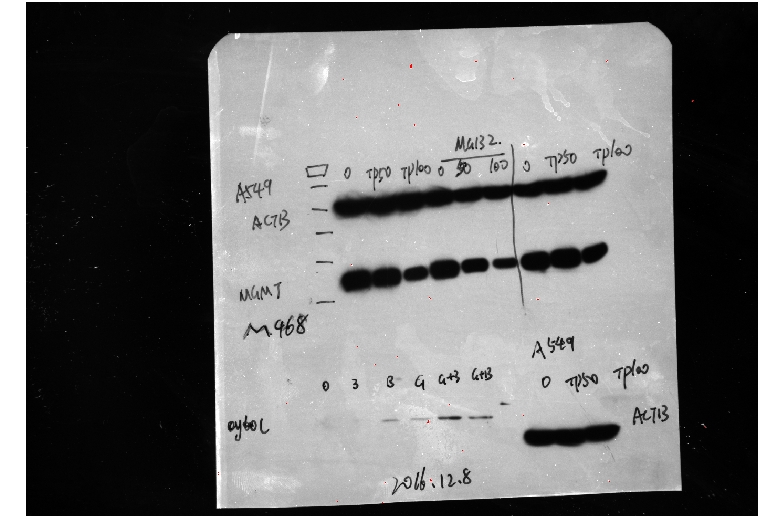


468 Cleaved caspase 3


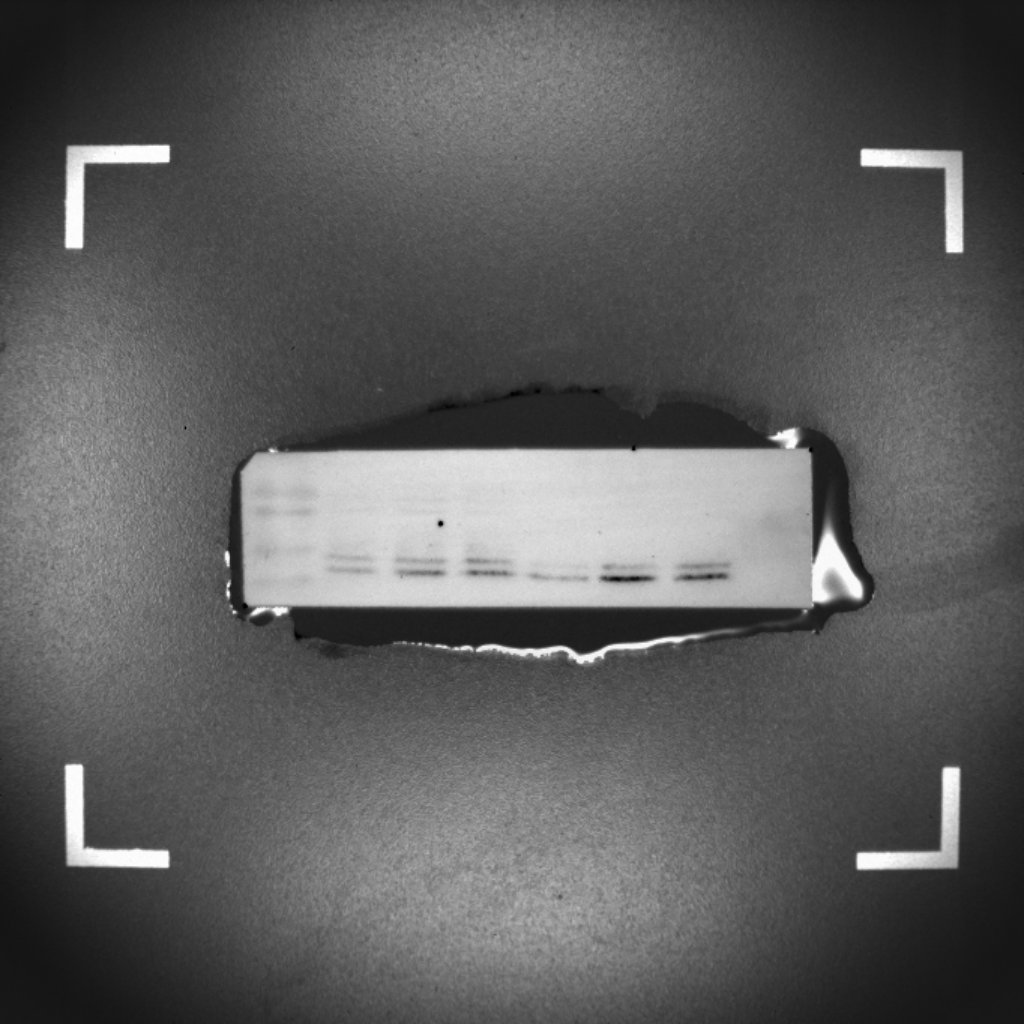


468 BAX


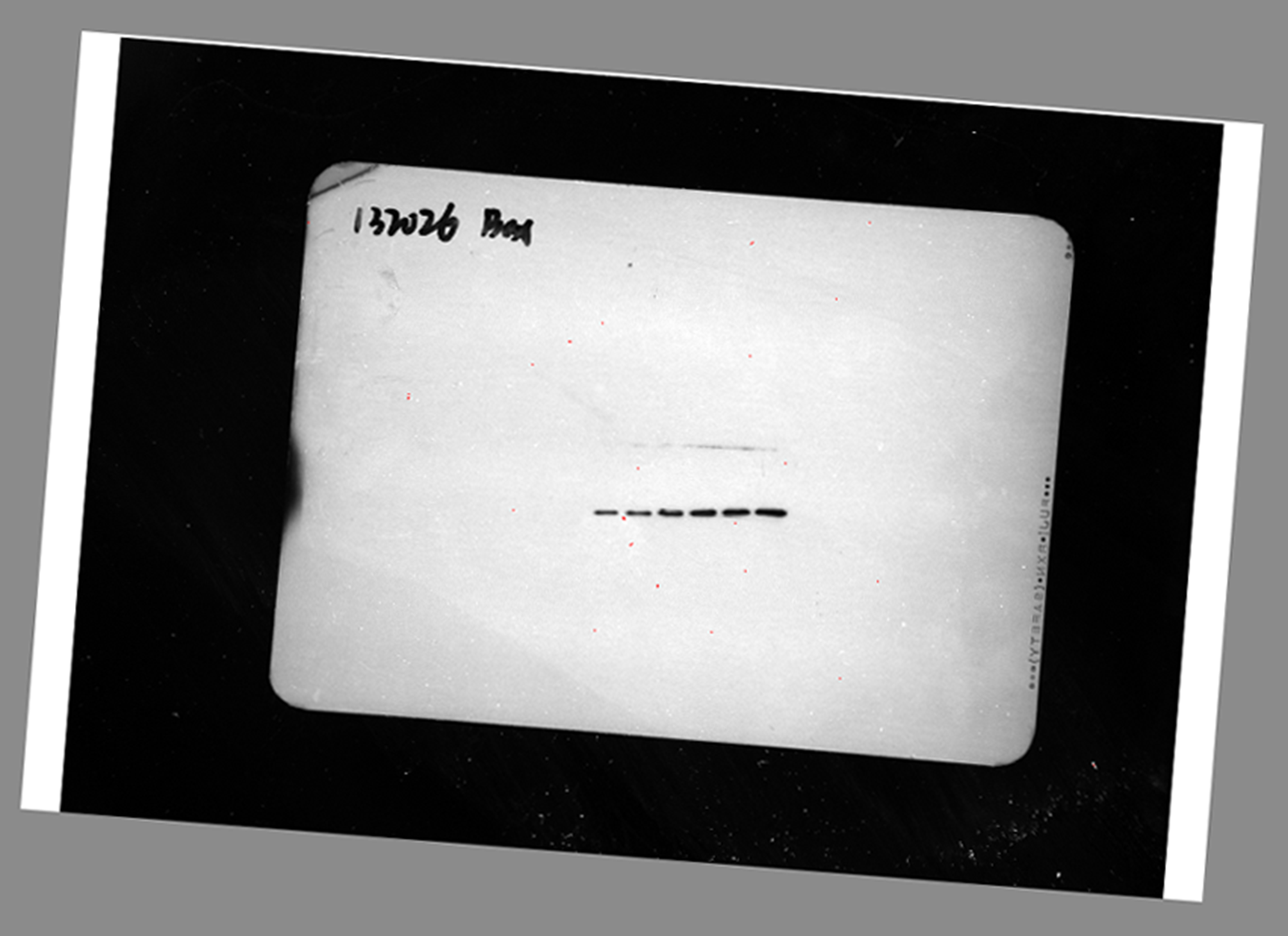


468 Bcl2


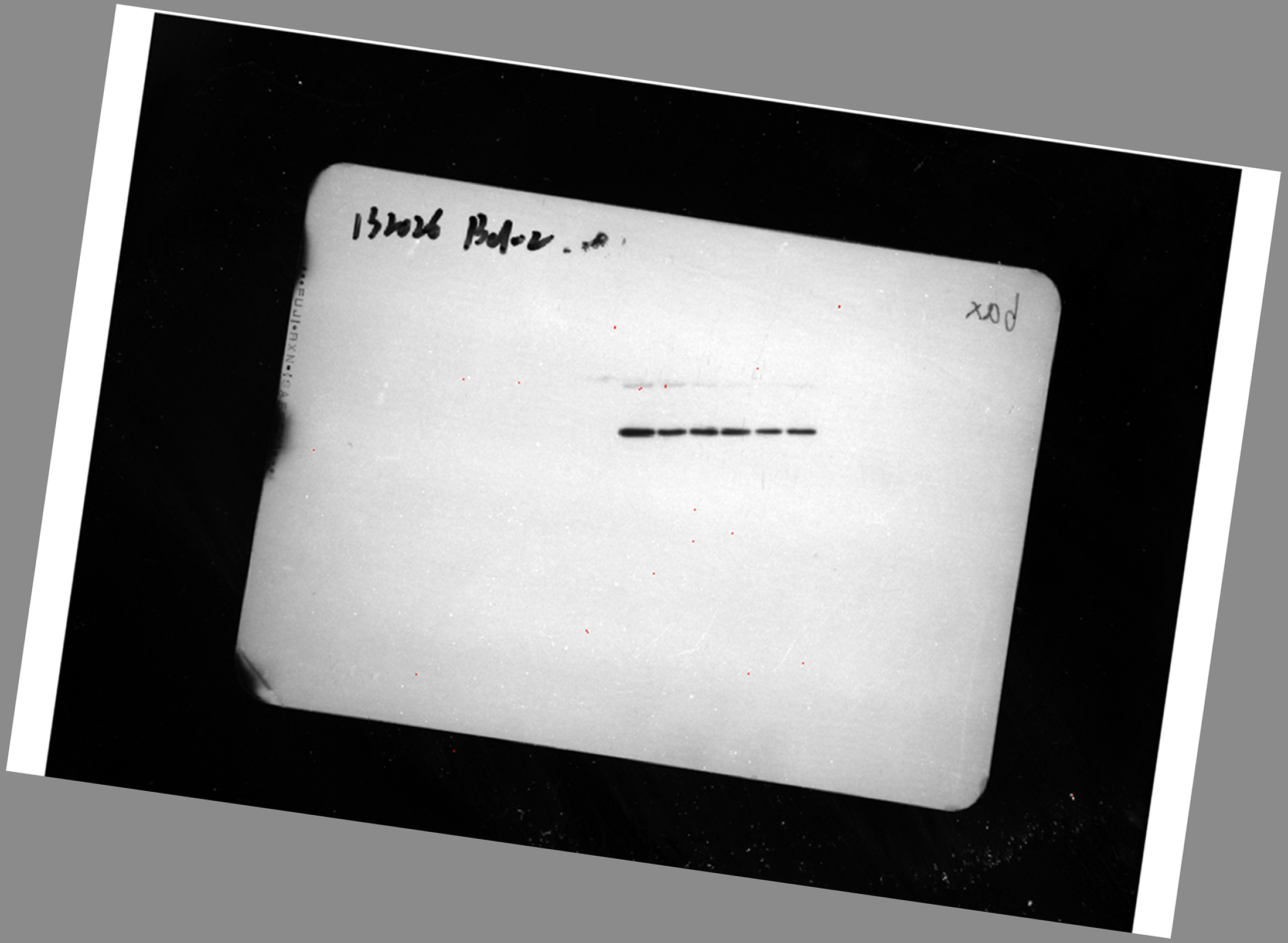


468 ACTB


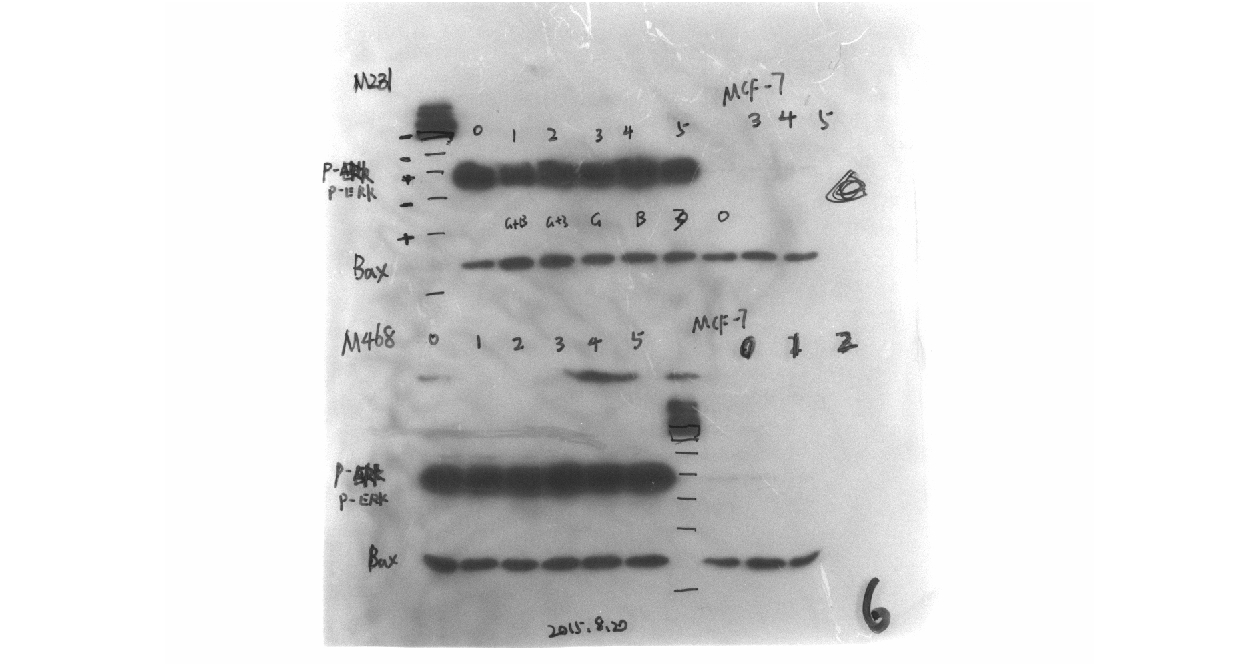

Supplement: S5 Dataset — (DOCX) [file pone.0177694.s005.docx]
